# Characterization of AP2/ERF and bHLH transcription factors and association with terpenoid metabolism in *Phlomoides rotata* from the Qinghai-Tibet Plateau

**DOI:** 10.3389/fpls.2026.1957015

**Published:** 2026-09-23

**Authors:** Rui Su, Xiaoli Yang, Yinglian Qi, Guigong Geng, Zirui Wang, Yaqi Li, Feng Qiao

**Affiliations:** 1 Key Laboratory of Tibetan Plateau Medicinal Plant and Animal Resources, School of Life Sciences, Qinghai Normal University, Xining, Qinghai, China; 2 College of Ecology and Environmental Science, Qinghai Institute of Technology, Xining, Qinghai, China; 3 Academy of Agricultural and Forestry Sciences, Qinghai University, Xining, China; 4 Academy of Plateau Science and Sustainability, Qinghai Normal University, Xining, Qinghai, China

**Keywords:** AP2/ERF, bHLH, bioinformatics, *Phlomoides rotata*, terpenoid metabolism, weighted gene co-expression network (WGCNA)

## Abstract

*Phlomoides rotata*, a Qinghai–Tibet Plateau endemic perennial valued for its hemostatic and anti-inflammatory effects, derives part of these bioactivities from terpenoid secondary metabolism. While AP2/ERF and bHLH transcription factors are established regulators of plant terpenoid biosynthesis, their roles in mediating *P. rotata*’s adaptation to natural high-altitude gradients remain uncharacterized. Here, we integrated transcriptomic and metabolomic profiles from four wild *P. rotata* populations spanning the 3,540-4,270 m altitudinal gradient to investigate the associations of AP2/ERF and bHLH with terpenoid biosynthesis. Genome-wide identification retrieved 169 AP2/ERF and 212 bHLH family members, of which 71 AP2/ERF and 50 bHLH genes displayed significant differential expression across four populations. Phylogenetic analysis resolved AP2/ERF members into 15 subfamilies and bHLH members into 21 subfamilies, including a *P. rotata*-specific clade (subgroup XXII). We further focused on differential expression of AP2/ERF and bHLH. Bioinformatic profiling revealed most encoded proteins are nuclear-localized, acidic, and unstable, with phytohormone-responsive cis-elements dominating AP2/ERF promoters (39.31%) and abiotic stress-responsive elements enriched in bHLH promoters (41.55%). Weighted gene co-expression network analysis (WGCNA) identified eight modules, with the green module showing the strongest correlation with terpenoid metabolites (12 of 52 significant associations, |*r*| > 0.7, *p* < 0.01). Intra-modular analysis revealed distinct correlation patterns: specific AP2/ERF and bHLH TFs exhibited strong positive correlations with iridoid glycosides, such as shanzhiside methyl ester, and key structural genes, including *IO* and *GGR*, but strong negative correlations with triterpenoid saponins, such as azukisaponin IV. qRT-PCR validation confirmed the reliability of the transcriptomic profiles. This study offers insights into the correlative framework linking AP2/ERF and bHLH transcription factors with terpenoid secondary metabolism in *P. rotata*.

## 1 Introduction

*Phlomoides rotata* (Benth. ex Hook.f.) Mathiesen (syn. *Lamiophlomis rotata* (Benth. ex Hook.f.) Kudô), a perennial herb endemic to the Qinghai–Tibet Plateau, is an important medicinal plant used in Tibetan and other regional medicines. Modern pharmacological investigations indicate that its bioactivity is closely associated with three dominant classes of specialized metabolites: iridoids (e.g., loganic acid, gentiopicroside), flavonoids (e.g., luteolin derivatives), and phenylethanoid glycosides (e.g., acteoside), which together are used to treat postoperative pain (Zhu et al., 2014), traumatic injuries (Lei et al., 2023; Yi et al., 2026), fractures (Gao et al., 2018), and bone pain (Zheng et al., 2015; Cui et al., 2020). Iridoid glycosides, a subclass of terpenoid secondary metabolites, represent the principal bioactive constituents of *P. rotata*. Notably, 8-O-acetylshanzhiside methyl ester and shanzhiside methyl ester serve as the core hemostatic components (Li et al., 2025c). Recent metabolomic studies have identified 26 iridoid compounds in *P. rotata*, highlighting the chemical diversity of its terpenoid profile (Zhang et al., 2018). Habitats across the Qinghai–Tibet Plateau exhibit wide elevational gradients, substantial temperature fluctuations, intense ultraviolet radiation, and pronounced spatial heterogeneity in precipitation. These long-term, complex environmental conditions have driven the differentiation of secondary metabolite profiles in *P*. rotata, resulting in marked variation in terpenoid accumulation among different wild populations (Zhong et al., 2025).

The biosynthesis of terpenoids is orchestrated by a complex regulatory network in which transcription factors (TFs) act as master switches (Liu et al., 2024). Among the plant transcription factor families, the AP2/ERF and bHLH families are pivotal in coordinating secondary metabolite accumulation. AP2/ERF is a plant-specific transcription factor family that accounts for 6–7% of all plant transcription factors and regulates downstream genes involved in terpenoid biosynthesis by directly binding to cis-regulatory elements such as the GCC-box, CE1, and DRE/CRT, or by physically interacting with other transcription factor families, including MYB, bHLH, and WRKY (Chen et al., 2026a). For example, AaERF1 and AaERF2 in *Artemisia annua* promote artemisinin biosynthesis by activating *ADS* and *CYP71AV1* (Yu et al., 2012). In *Litsea cubeba*, LcERF19 enhances geranial and neral biosynthesis through activation of *LcTPS42* expression, which provides a strategy to improve the flavor of tomato and other fruits (Wang et al., 2022). *SmERF128* in *Salvia miltiorrhiza* promotes tanshinone biosynthesis by upregulating *SmCPS1* (Zhang et al., 2019). Similar to AP2/ERF transcription factors, bHLH transcription factors also serve as central regulatory nodes in terpenoid metabolism. Through their conserved basic helix–loop–helix transcriptional activation domain, bHLH proteins bind to E-box (CANNTG) elements and form homo- or heterodimers, thereby activating genes encoding key enzymes involved in terpenoid biosynthesis. For example, in tomato, jasmonate-induced *SlJIG* is a direct target of *MYC2* and forms a *MYC2–SlJIG* regulatory module that promotes terpene biosynthesis by upregulating terpene synthase (TPS) genes, thereby enhancing resistance to cotton bollworm and *Botrytis cinerea* (Zhang et al., 2025). Overexpression of *LaMYC4* in *Lavandula angustifolia* markedly increases volatile terpenoid production (Dong et al., 2022), and overexpression of *AabHLH112* in *Artemisia annua* elevates β-caryophyllene content (Xiang et al., 2022). AP2/ERF and bHLH transcription factors can also form synergistic regulatory complexes that jointly activate terpenoid biosynthesis. A study published in The Crop Journal provided the first evidence that, in Chinese prickly ash (*Zanthoxylum bungeanum*), the ZbERF6–ZbbHLH2 complex significantly promotes monoterpene accumulation in the pericarp by synergistically activating the expression of *ZbIDI*, which encodes a rate-limiting enzyme in monoterpene biosynthesis (Zhang et al., 2026a). This finding is highly consistent with the “co-regulatory paradigm” in which AP2/ERF and bHLH transcription factors regulate plant specialized metabolism through protein–protein interactions (Chen et al., 2026a), suggesting that the AP2/ERF–bHLH module may represent a conserved regulatory node in the transcriptional control of terpenoid biosynthesis across diverse medicinal plant species. In addition to TF-mediated regulation, environmental factors also play a critical role in terpenoid biosynthesis. For example, high light promotes diterpenoid lactone accumulation in *Andrographis paniculata* (Zhou et al., 2026), illustrating that environmental cues drive terpenoid metabolism in medicinal plants. However, although the pivotal regulatory roles of AP2/ERF and bHLH transcription factors in the biosynthesis of high-value terpenoids—including artemisinin, tanshinones, monoterpenes, and diterpenes have been extensively characterized, genome-wide identification and phylogenetic characterization of these two transcription factor families in *P. rotata*, as well as analyses of their associations with iridoid metabolism, have not yet been reported. An integrated transcriptomic and metabolomic study published in *Plants* identified 575 metabolites in the leaves of *P. rotata*, including 455 differentially accumulated metabolites, and further identified eight core differential metabolites associated with iridoid metabolism: shanzhiside methyl ester, 8-epi-deoxyloganic acid, barlerin, shanzhiside, geniposide, agnuside, feretoside, and catalpin (Wang et al., 2024). Nevertheless, that study did not systematically investigate the transcription factors governing the accumulation of these iridoids.

Weighted gene co-expression network analysis (WGCNA) provides a powerful systems biology tool for linking transcription factors to metabolic phenotypes. This approach has been successfully applied to construct terpenoid regulatory networks in various plant species. For example, in *finger citron*, WGCNA identified modules linking TFs such as AP2/ERF, bHLH, and MYB with volatile terpenoids (Xu et al., 2019). In the flowers of *Dendrobium officinale*, WGCNA revealed significant correlations between 13 terpene synthase genes and five transcription factor families—AP2/ERF, bHLH, WRKY, MYB, and bZIP (Li et al., 2021). In grapevine, WGCNA identified the MEblue module as the module most strongly associated with monoterpene content and predicted *VvbHLH02* as a potential transcriptional regulator (Wang et al., 2026). Collectively, these studies demonstrate that WGCNA-based integration of transcriptomic and metabolomic data represents a robust strategy for identifying transcription factor–metabolite regulatory modules within complex phytochemical pathways.

Against this background, wild populations of *P*. rotata distributed along an altitudinal gradient of 3,540-4,270 m on the Qinghai–Tibet Plateau were investigated to explore, for the first time, the associations of the AP2/ERF and bHLH transcription factor families with terpenoid secondary metabolism. Specifically, we performed global transcriptomics analyses of *P*. rotata leaves collected from four elevations (HN, 3,540 m; GL, 3,750 m; YS, 3,880 m; and CD, 4,270 m) to identify the AP2/ERF and bHLH gene families at the genome-wide level and characterize their phylogenetic relationships, chromosomal distributions, gene duplication patterns, protein physicochemical properties, and cis-acting elements. We employed WGCNA to construct co-expression modules linking transcription factor expression, the expression of terpenoid biosynthetic genes, and the abundances of 38 annotated metabolites.

## 2 Materials and methods

### 2.1 Plant materials

*P. rotata* was collected from four wild populations located in distinct geographical regions of Qinghai Province, covering an altitude range of 3540 to 4270 meters (**Table 1**). In July, leaves from healthy, uniformly growing, pest-free plants were collected, surface-sterilized, quickly frozen in liquid nitrogen, and stored at -80 °C for subsequent experiments.

**Table 1:** *P. rotata* samples from four different habitats.

Symbol	HN	GL	YS	CD
Location	Henan County	Guoluo Prefecture	Yushu Prefecture	Chengduo County
Altitude	3,540 m	3,750 m	3,880 m	4,270 m
East longitude	101°56′48″	100°14′38″	97°1′23″	97°27′16″
North latitude	34°16′12″	34°29′10″	32°51′4″	33°18′2″
Mean annual precipitation	579.7 mm	513.5 mm	500.0 mm	459.0 mm
Mean annual temperature	0.3 °C	-0.5 °C	-3.6 °C	-1.6 °C
Mean annual sunshine duration	2,537 h	2,570 h	2,477 h	2,617 h
Mean annual evaporation	1,193.0 mm	1,322.1 mm	1,259.1 mm	1,688.0 mm

### 2.2 Differentially expressed genes (DEGs) analysis

RNA-seq raw reads were obtained from our research group’s previously published datasets (NCBI SRA under the project number: PRJNA1076649 [https://www.ncbi.nlm.nih.gov/sra/PRJNA1076649]) (Li et al., 2024b). All downstream processing was performed independently in this study. During data quality control, reads containing adapter-containing reads and low-quality reads were removed. Transcriptome sequencing data quality was assessed by analyzing base quality distribution, base content distribution, and average read quality. In previously published analyses from our research group, transcripts were primarily assembled *de novo* using Trinity because a reference genome was not available. In the present study, as a chromosome-level reference genome for *P. rotata* has now been published (https://ftp.ncbi.nlm.nih.gov/genomes/all/GCA/051/938/595/) (Tian et al., 2025), the dataset was reanalyzed using a reference-guided strategy. Clean reads were aligned to the reference genome with HISAT2, and BAM files were generated. Based on the exon structures defined in the annotated GTF file, HTSeq (Putri et al., 2022) was used to calculate the read count for each gene as the raw expression value. These values were then normalized using FPKM (Fragments Per Kilobase of transcript per Million fragments mapped), which served as an indicator of gene expression level. Sample-to-sample Pearson correlation coefficients and principal component analysis (PCA) were used to assess overall expression patterns and intergroup differences. Differential expression analysis was performed using DESeq2 (Love et al., 2014) to identify differentially expressed genes (DEGs). DEGs were identified using the thresholds of |log2FoldChange| ≥ 2 and P-value < 0.01, followed by GO (Gene Ontology; The Gene Ontology Consortium et al., 2026) and KEGG (Kyoto Encyclopedia of Genes and Genomes; Kanehisa et al., 2025) annotation.

### 2.3 Identification of differential TFs

The *P. rotata* genome file was downloaded from NCBI (National Center for Biotechnology Information; https://ftp.ncbi.nlm.nih.gov/genomes/all/GCA/051/938/595/) (Tian et al., 2025). Reference protein sequences for plant TFs were obtained from the PlantTFDB (Release v5.0, https://planttfdb.gao-lab.org) (Tian et al., 2019). BLASTp was used for homologous sequence alignment under the condition of E-value, and the identified TFs were classified into families. InterProScan-5.76-107.0 (Blum et al., 2025) was used for protein domain verification of the filtered results. Entries with an E-value of, and sequences with incomplete domains were removed. The Swiss-Prot (The UniProt Consortium et al., 2025) database was used for functional annotation, removing annotations of non-transcription factor sequences. By integrating genome-wide TF predictions with DEGs from the transcriptome samples, we identified differentially expressed TFs, with a particular focus on the AP2/ERF and bHLH families for subsequent analyses. Protein and CDS sequences of AP2/ERF and bHLH TFs are listed in **Supplementary Tables 1**, **2**.

### 2.4 Chromosomal distribution, duplication events, and phylogenetic analysis

A total of 169 AP2/ERF and 212 bHLH family members were retrieved from the genome annotation and mapped to chromosomes. Chromosomal distribution was visualized using TBtools (Chen et al., 2020, 2023). Gene duplication events and syntenic relationships were analyzed using MCScanX (Wang et al., 2012) based on sequence similarity and physical positions; collinearity and chromosomal locations were further illustrated using the Advanced Circos tool implemented in TBtools.

For phylogenetic analysis, reference protein sequences of AP2/ERF and bHLH TFs from *Arabidopsis thaliana* were retrieved from PlantTFDB (http://planttfdb.gao-lab.org/) and combined with the 169 AP2/ERF and 212 bHLH sequences identified in this study. Redundant suffixes in protein IDs were trimmed to retain unique identifiers. Multiple sequence alignments were generated using MAFFT v7.526 (Rozewicki et al., 2019) with default parameters. Maximum likelihood phylogenetic trees were constructed using IQ-TREE v2.2.6 (Nguyen et al., 2015) with 1000 ultrafast bootstrap replicates to assess node support. Trees were visualized and annotated using iTOL v6 (Letunic and Bork, 2024).

### 2.5 Bioinformatic characterization of AP2/ERF and bHLH TFs

The physicochemical properties, including parameters such as amino acid count, molecular weight, theoretical isoelectric point (pI), instability index (II), aliphatic index (AI), and grand average of hydropathicity (GRAVY), were determined for the AP2/ERF and bHLH differentially expressed proteins via the ExPASy ProtParam (https://web.expasy.org/protparam) server (Gasteiger et al., 2005). Additionally, subcellular localization was predicted using DeepLoc 2.0 (https://services.healthtech.dtu.dk/services/DeepLoc- 2.0/) (Thumuluri et al., 2022).

The Conserved Domain Database from the National Center for Biotechnology Information (NCBI Batch CD-search) (Wang et al., 2023) was used to identify and confirm the conserved domains of TFs. The protein sequences of the differentially expressed AP2/ERF and bHLH TFs were submitted to MEME Suite (https://meme-suite.org/) (Bailey et al., 2015) to identify motif distribution patterns. Gene structure annotation information was extracted from the downloaded genome GFF annotation file. Conserved domains, motif composition, and gene structures were visualized using the Gene Structure View module in TBtools (Chen et al., 2020, 2023).

To investigate cis-acting elements in the promoter regions of AP2/ERF and bHLH genes in *P. rotata* leaves, the 2,000-bp sequences upstream of the ATG start codon were defined as putative promoter regions and were extracted using the Fasta Extract tool in TBtools. The extracted promoter regions were analyzed using the PlantCARE database (Lescot, 2002) to identify putative cis-regulatory elements, excluding core promoter elements required for basal transcription in higher plants, such as TATA boxes. Promoter-associated cis-acting elements were visualized using R (version 4.5.2).

### 2.6 Terpenoid metabolite extraction and quantification

Metabolites were extracted and analyzed using a Waters Acquity I-Class PLUS UPLC system coupled to an Applied Biosystems QTRAP 6500+ mass spectrometer. Chromatographic separation was performed on a Waters HSS-T3 column (1.8 µm, 2.1 × 100 mm) with a gradient elution of (A) water (0.1% formic acid, 5 mM ammonium acetate) and (B) acetonitrile (0.1% formic acid) at 0.35 mL/min. The column oven was maintained at 50 °C. Mass spectrometric detection was conducted using an electrospray ionization (ESI) source in both positive and negative ion modes (5500 V/−4500 V). Data acquisition was performed via Multiple Reaction Monitoring (MRM). Raw data were processed using MultiQuant™, and peak areas were normalized to the total peak area. Principal component analysis (PCA) and Spearman correlation were employed to assess sample repeatability. Orthogonal Partial Least Squares-Discriminant Analysis (OPLS-DA) was conducted using the ropls package in R, with 200 permutations validating the model. Differential terpenoid metabolites were screened based on the following criteria: fold change (|log2FC) > 1, variable importance in projection (VIP) > 1, and p-value < 0.05 (t-test). Metabolite abundances are listed in **Supplementary Table 3**.

### 2.7 Weighted gene co-expression network analysis (WGCNA)

Differentially expressed AP2/ERF and bHLH transcription factors, alongside KEGG-annotated structural genes involved in terpenoid biosynthesis, were extracted from transcriptomes of four *P. rotata* populations. Raw gene counts were imported and filtered to remove genes with zero reads across all samples. Given the focused nature of this study on a predefined set of 159 metabolic genes and the limited sample size (*n* = 12), stringent filtering based on counts per million (CPM) was relaxed to avoid excluding biologically relevant low-abundance transcripts. Library sizes were normalized using the Trimmed Mean of M-values (TMM) method implemented in the edgeR package (v3.42.0) to correct for compositional biases. To transform the count data into a format suitable for WGCNA, which assumes a normally distributed input, the voom function from the limma package (v3.56.0) was applied. This procedure models the mean-variance relationship and converts raw counts into precision-weighted log2-counts per million (logCPM) values, referred to as VST (Variance Stabilized Transformed) data (**Supplementary Table 4**). WGCNA was performed using the R package WGCNA, with terpenoid secondary metabolite abundances set as phenotypic traits. Metabolite abundance data were preprocessed as follows. The predefined value 0.000167923 was treated as an undetected signal (missing value). Metabolites with undetected signals in ≥50% of samples were discarded. Remaining undetected values were replaced with NA. The filtered abundance matrix was subjected to log2(x+1) transformation to normalize data distribution for subsequent analysis (**Supplementary Table 3**). Due to the limited sample size (*n* = 12), the scale-free topology fit index (R²) did not reach the conventional threshold of 0.85 even at high soft-threshold powers. Therefore, a soft-thresholding power of *β* = 6 was selected to meet the scale-free topology criterion for the 12-sample dataset. Genes were clustered using dynamic tree cutting (minimum module size = 10). Modules with eigengene correlations > 0.85 (dissimilarity < 0.15) were merged to reduce redundancy. Associations between module eigengenes and 38 core terpenoid metabolites were assessed using biweight mid-correlation (bicor). A stringent threshold of *|r*| > 0.7 (*p* < 0.01) was applied to identify robust module-metabolite relationships. Subsequently, an expression pattern analysis was performed on the robust module-metabolite relationships with the highest number of metabolites.

### 2.8 Quantitative real-time PCR (qRT-PCR) validation

Primers for RT-qPCR were designed using Premier 6 to validate the expression patterns of selected differentially expressed TFs and thereby assess the reliability of the transcriptome data. All primer sequences were synthesized by Qingke Biotechnology Co., Ltd. (Xi’an, China). Total RNA was extracted from *P. rotata* leaves using the SPARKeasy Plant RNA Kit (SparkJade Biotechnology Co., Ltd., Shandong, China), and RNA quality and concentration were evaluated by 1.2% agarose gel electrophoresis and a BioSpec-nano UV–visible spectrophotometer, respectively. First-strand cDNA was synthesized using the SPARKscript II All-in-One RT SuperMix for qPCR with gDNA Eraser (SparkJade Biotechnology Co., Ltd., Shandong, China) and stored at −20 °C until use. RT-qPCR was performed using 2× Universal SYBR Green qPCR Mix on a Bio-Rad iQ5 system in a 20 μL reaction containing 10 μL SYBR mix, 1 μL of 5-fold diluted cDNA template (equivalent to 200 ng), 0.4 μL each of forward and reverse primers, and RNase-free water to bring the total volume to 20 μL. The amplification program consisted of an initial denaturation at 95 °C for 30 s, followed by 40 cycles of 95 °C for 10 s and 60 °C for 30 s, with melt-curve analysis performed at the end of the run. Negative controls were included, and each sample was analyzed with three biological replicates. Relative gene expression levels were calculated using the 2^-ΔΔCt^ method (Livak and Schmittgen, 2001).

## 3 Results

### 3.1 Global transcriptomics in *P. rotata* leaves across four wild populations

The reference genome alignment results for wild *P. rotata* samples were presented in **Supplementary Table 5**. Although sequence alignment rates varied among populations, the gene coverage curves of all 12 samples exhibited a typical bell-shaped distribution, with high coverage across the main gene body regions (> 0.9) (**Supplementary Figure 1**). These results indicated that, despite relatively low uniquely mapped rates in some samples, the sequencing data provided sufficient depth and uniformity for reliable quantification of gene expression. To assess biological reproducibility and validate sample grouping, Pearson correlation analysis was performed based on the FPKM (Fragments Per Kilobase of transcript per Million mapped reads) values of the 12 samples (**Figure 1A**). Samples from the same region showed very strong positive correlations (*r* > 0.95), with the HN group exhibiting an intragroup correlation as high as 0.97, indicating excellent biological reproducibility. Clear clustering patterns were observed among populations, suggesting marked differences in leaf transcriptomic profiles of *P. rotata* across the four regions. Principal component analysis (PCA) of the gene expression matrix further supported this pattern (**Figure 1B**). PC1 and PC2 explained 50.10% and 24.63% of the total variance, respectively, with a cumulative contribution of 74.73%, indicating that the first two principal components captured most of the variation among samples. In the two-dimensional PCA space, the four population groups (HN, GL, YS, and CD) formed distinct clusters and were clearly separated from one another.

**Figure 1:**
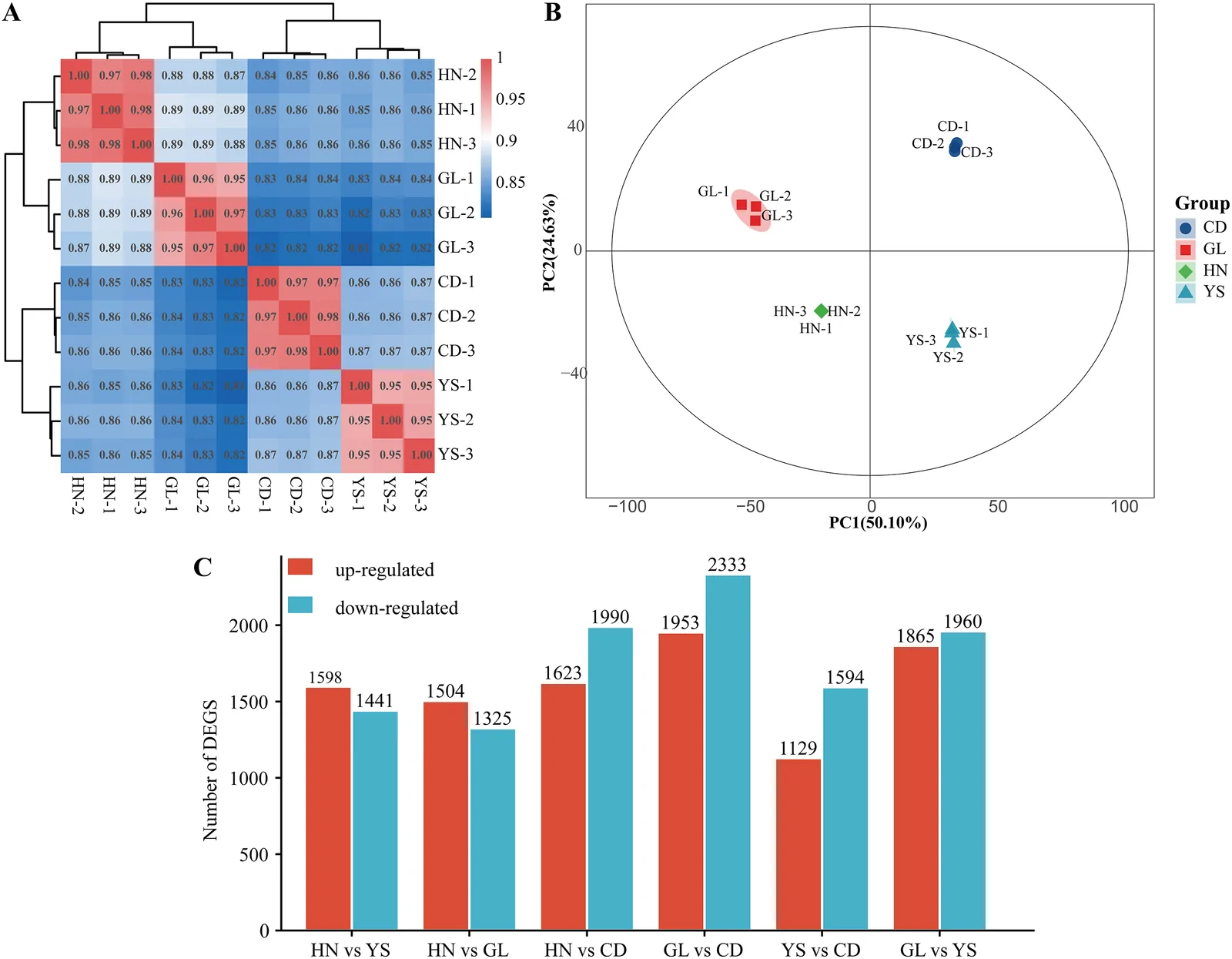
Transcriptome analysis of *P. rotata* leaves from four regions at elevations ranging from 3,540 to 4,270 (m). **(A)** Pearson correlation matrix of gene expression profiles. **(B)** Principal component analysis (PCA). **(C)** Numbers of upregulated and downregulated DEGs. HN, 3,540 m; GL, 3,750 m; YS, 3,880 m; CD, 4,270 m.

Differential expression analysis revealed substantial transcriptional divergence among populations (**Figure 1C**). A total of 3,039 differentially expressed genes (DEGs) were detected between HN and YS, including 1,598 upregulated and 1,441 downregulated genes. Between HN and GL, 2,829 DEGs were identified, of which 1,504 were upregulated and 1,325 were downregulated. In the HN vs. CD comparison, 3,603 DEGs were detected, including 1,613 upregulated and 1,990 downregulated genes. The GL vs. CD comparison yielded the largest number of DEGs (4,286), including 1,953 upregulated and 2,333 downregulated genes. In the YS vs. CD comparison, 2,723 DEGs were identified, including 1,129 upregulated and 1,594 downregulated genes. In addition, 3,825 DEGs were detected between GL and YS, including 1,865 upregulated and 1,960 downregulated genes. DEG counts did not increase monotonically with elevational distance, peaking at the GL vs. CD contrast (4,286 DEGs; 520 m apart) rather than the maximal HN vs. CD span (3,603 DEGs; 730 m apart).

### 3.2 GO and KEGG analysis of DEGs in *P. rotata* leaves

To characterize the functional divergence among the four wild populations, Gene Ontology (GO) and Kyoto Encyclopedia of Genes and Genomes (KEGG) enrichment analyses were performed using DEGs identified from the six pairwise comparisons (**Supplementary Tables 6**, **7**). In total, 139 GO terms and 69 KEGG pathways were significantly enriched across the comparisons (p.adjust < 0.05).

GO Biological Process (BP) enrichment analysis revealed both shared and comparison-specific functional signatures, with ‘secondary metabolite biosynthetic process’ and ‘secondary metabolic process’ consistently enriched across all population comparisons (**Figure 2**; full results in **Supplementary Table 6**). The HN vs GL comparison was characterized by significant enrichment in response to jasmonic acid (**Figure 2A**; **Supplementary Table 6**) and monooxygenase/oxidoreductase activities (GO Molecular Function; **Supplementary Table 6**), consistent with active terpenoid biosynthesis and modification. Notably, suberin and cutin biosynthesis was exclusively enriched in the HN vs GL and GL vs YS comparisons (RichFactor = 4.59 and 4.46, respectively), with no detectable enrichment in comparisons involving the CD population. In contrast, photosystem I was the most significantly enriched category in the GL vs CD comparison (RichFactor = 12.47) (GO Cellular Component; **Supplementary Table 6**). The YS vs CD comparison lacked specific enrichment of terpenoid-related terms but exhibited significant enrichment in purine metabolism (RichFactor = 2.29) and defense responses, highlighting a distinctive metabolic profile in this specific population pair.

**Figure 2:**
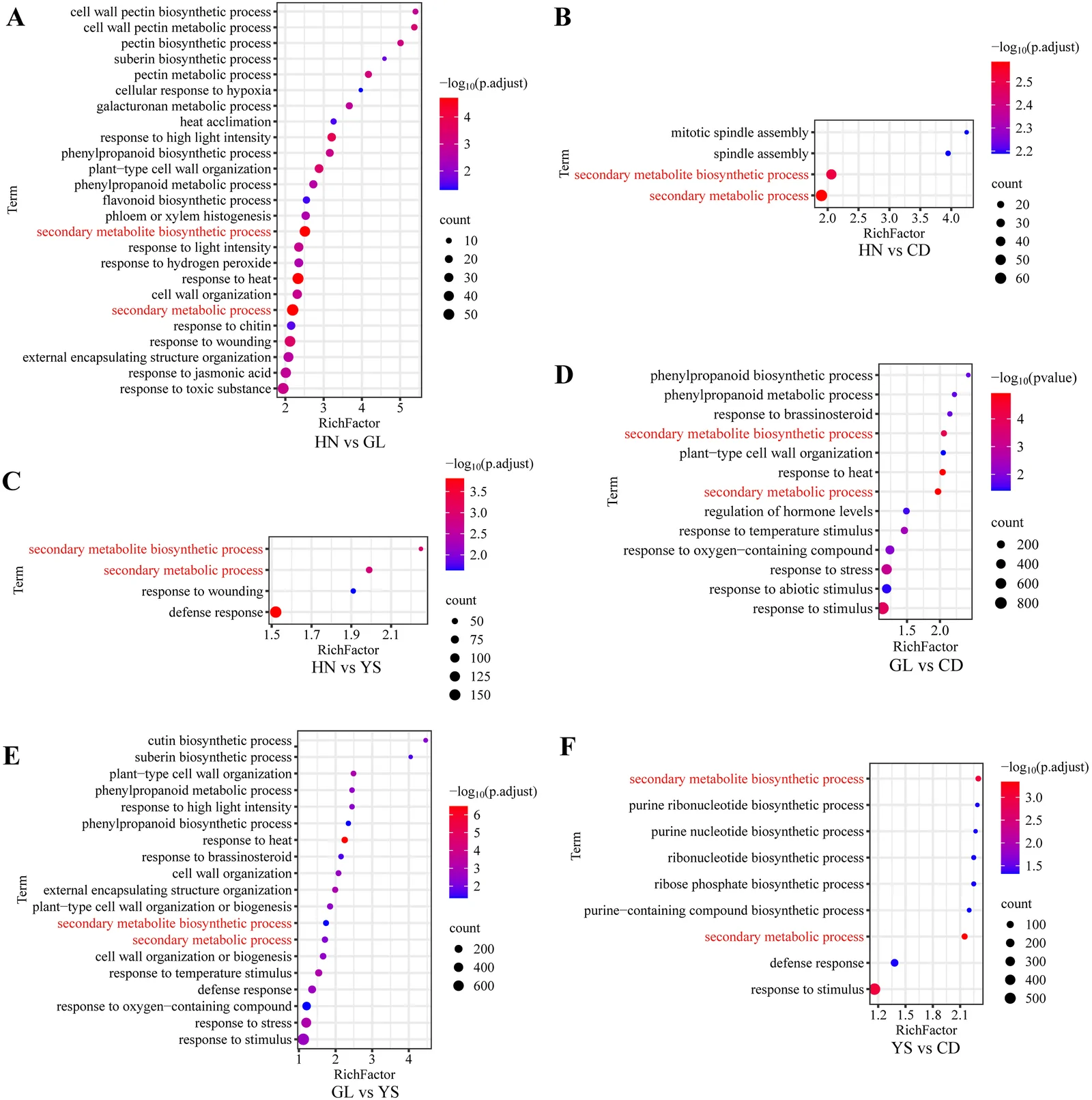
GO enrichment analysis of *P. rotata* leaves from four regions at elevations ranging from 3,540 to 4,270 m. **(A)** HN vs GL; **(B)** HN vs CD; **(C)** HN vs YS; **(D)** GL vs CD; **(E)** GL vs YS; **(F)** YS vs CD. HN: 3,540 m; GL: 3,750 m; YS: 3,880 m; CD: 4,270 m. The figure only shows biological process terms. The top 25 enriched BP terms are shown for HN vs GL (which had 44 total enriched terms); all terms are shown for comparisons with ≤25 enriched terms. Full results in **Supplementary Table 6**.

KEGG pathway analysis revealed distinct enrichment patterns of terpenoid biosynthetic pathways across the altitudinal comparisons. Monoterpenoid biosynthesis was most strongly enriched in the HN vs CD group (Rich Factor = 3.53), followed by HN vs YS (Rich Factor = 3.22), HN vs GL (Rich Factor = 3.09), and GL vs YS (Rich Factor = 3.07), with the weakest enrichment detected in GL vs CD (Rich Factor = 2.27). Terpenoid backbone biosynthesis was restricted exclusively to HN vs YS (RF = 2.10) and HN vs CD (Rich Factor = 1.94). Sesquiterpenoid and triterpenoid biosynthesis was specifically enriched in HN vs YS (Rich Factor = 2.49) and HN vs GL (Rich Factor = 2.35) (**Figure 3**).

**Figure 3:**
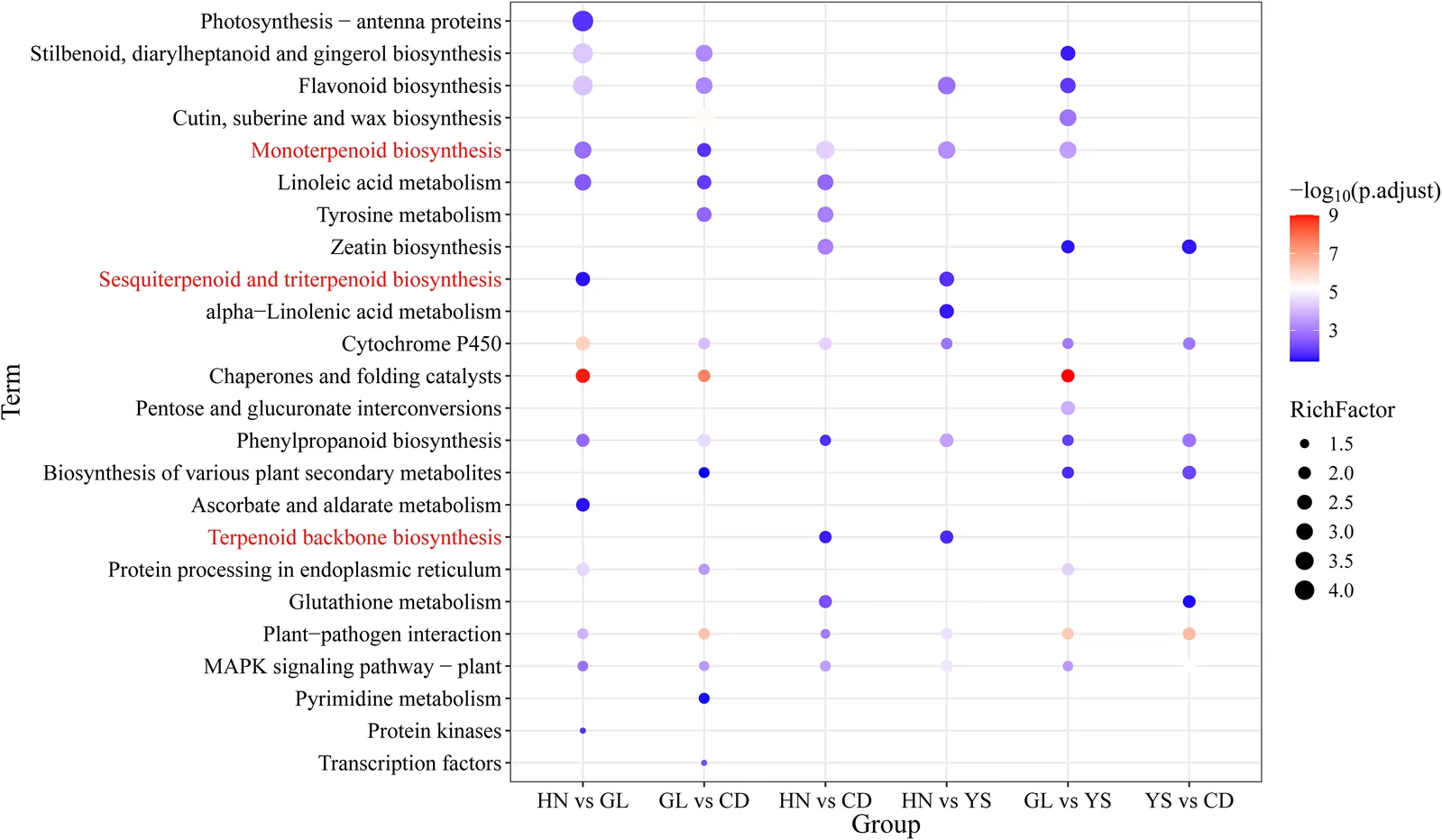
KEGG pathway enrichment analysis of *P. rotata* leaves from four regions at elevations ranging from 3,540 to 4,270 m. HN: 3,540 m; GL: 3,750 m; YS: 3,880 m; CD: 4,270 m. Full results in **Supplementary Table 7**.

### 3.3 Identification and screening of differentially expressed AP2/ERF and bHLH transcription factors

Genome-wide homology searches against *Arabidopsis thaliana* transcription factor datasets identified 54 TF families in *P. rotata* leaves. Using the thresholds of |log2FoldChange| ≥ 2 and P-value < 0.01, differentially expressed TFs (DE-TFs) were detected in 42 of these families (**Figure 4**; **Supplementary Table 8**). The five largest TF families were FAR1 (244 members), bHLH (212 members), AP2/ERF (169 members; comprising AP2, ERF, RAV, and DREB subfamilies), bZIP (152 members), and MYB (145 members). Notably, the 71 DE-TFs of AP2/ERF and 50 DE-TFs of bHLH represent the two largest pools of differentially expressed transcription factors among all families in *P. rotata*. In contrast, twelve families—including VOZ, HB-other, NF-YC, BES1, LSD, CAMTA, GeBP, S1Fa-like, Whirly, HB-PHD, HRT-like, and LFY—exhibited no differential expression across the population comparisons.

**Figure 4:**
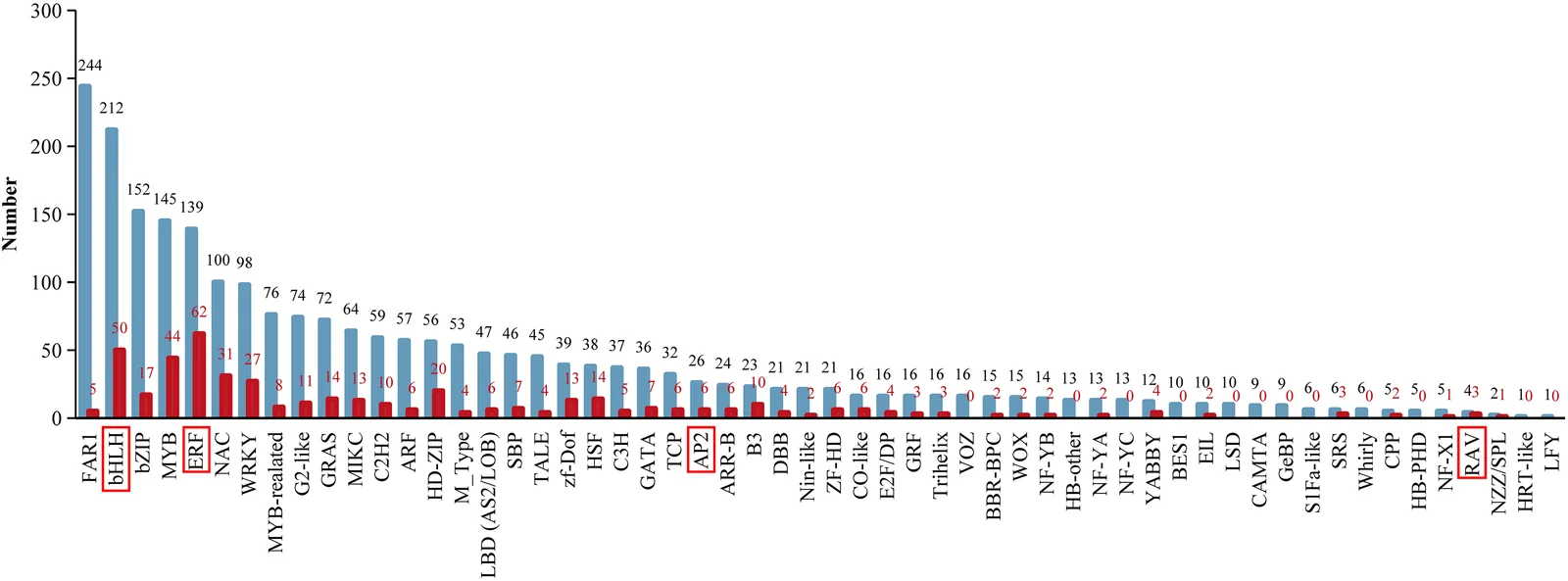
The analysis identified 54 distinct TF families (blue), of which 42 were differentially expressed (red) in *P. rotata* leaves.

Given the substantial number of DE-TFs in the AP2/ERF and bHLH families, these two families were selected for subsequent in-depth analysis. A total of 71 nonredundant AP2/ERF genes and 50 nonredundant bHLH genes possessing complete open reading frames (ORFs) were ultimately retained for downstream characterization. Detailed annotations are provided in **Supplementary Table 9**.

### 3.4 Genomic architecture of the AP2/ERF and bHLH gene families

Maximum-likelihood phylogenetic trees were constructed using AP2/ERF and bHLH protein sequences from *P. rotata* and *Arabidopsis thaliana*. Based on the established classification in *A. thaliana*, the identified AP2/ERF genes in *P. rotata* were categorized into AP2, DREB, ERF, RAV, and Soloist. Consistent with previous classifications, the DREB and ERF subfamilies were further subdivided into the A1–A6 and B1–B6 groups, respectively (Nakano et al., 2006; **Figure 5**). Phylogenetic analysis revealed that *P. rotata* members were distributed across all subfamilies. The B-3 subgroup of the ERF branch constituted the largest clade, comprising 32 genes, the majority of which formed a distinct and well-supported monophyletic clade. Similarly, the bHLH phylogenetic analysis assigned the identified genes to 20 of the 21 established subgroups (I–XXI), with no representatives detected in subgroup XX (**Figure 6**). Additionally, a distinct and well-supported clade comprising eight *P. rotata*-specific bHLH members was identified and designated as subgroup XXII (Toledo-Ortiz et al., 2003; **Figure 6**).

**Figure 5:**
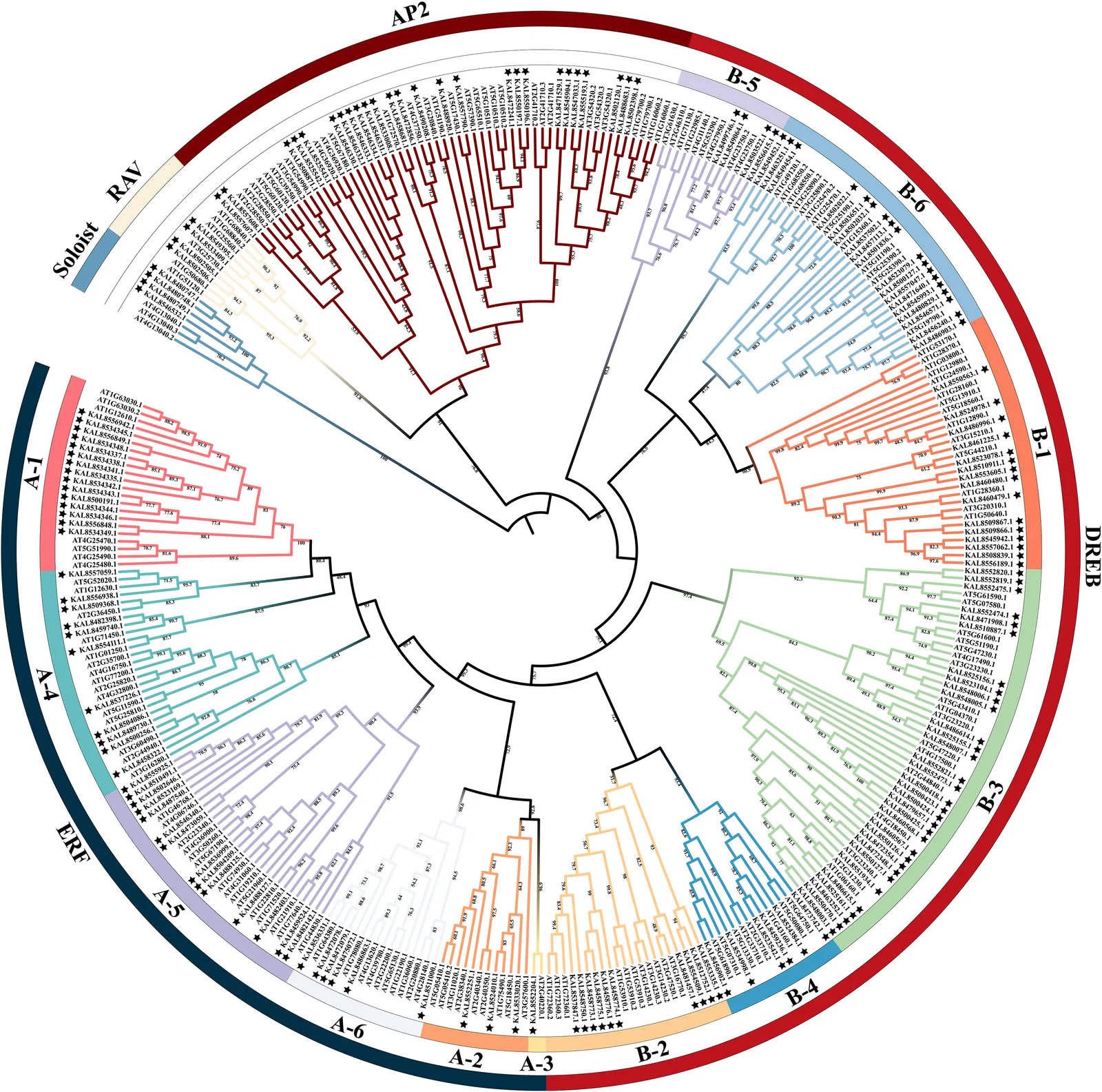
Phylogenetic reconstruction of AP2/ERF TF genes in *P. rotata* (★) and *A. thaliana*. Bootstrap values > 40% are shown at the corresponding nodes. The resulting phylogeny resolved the AP2/ERF TF into 15 distinct clades.

**Figure 6:**
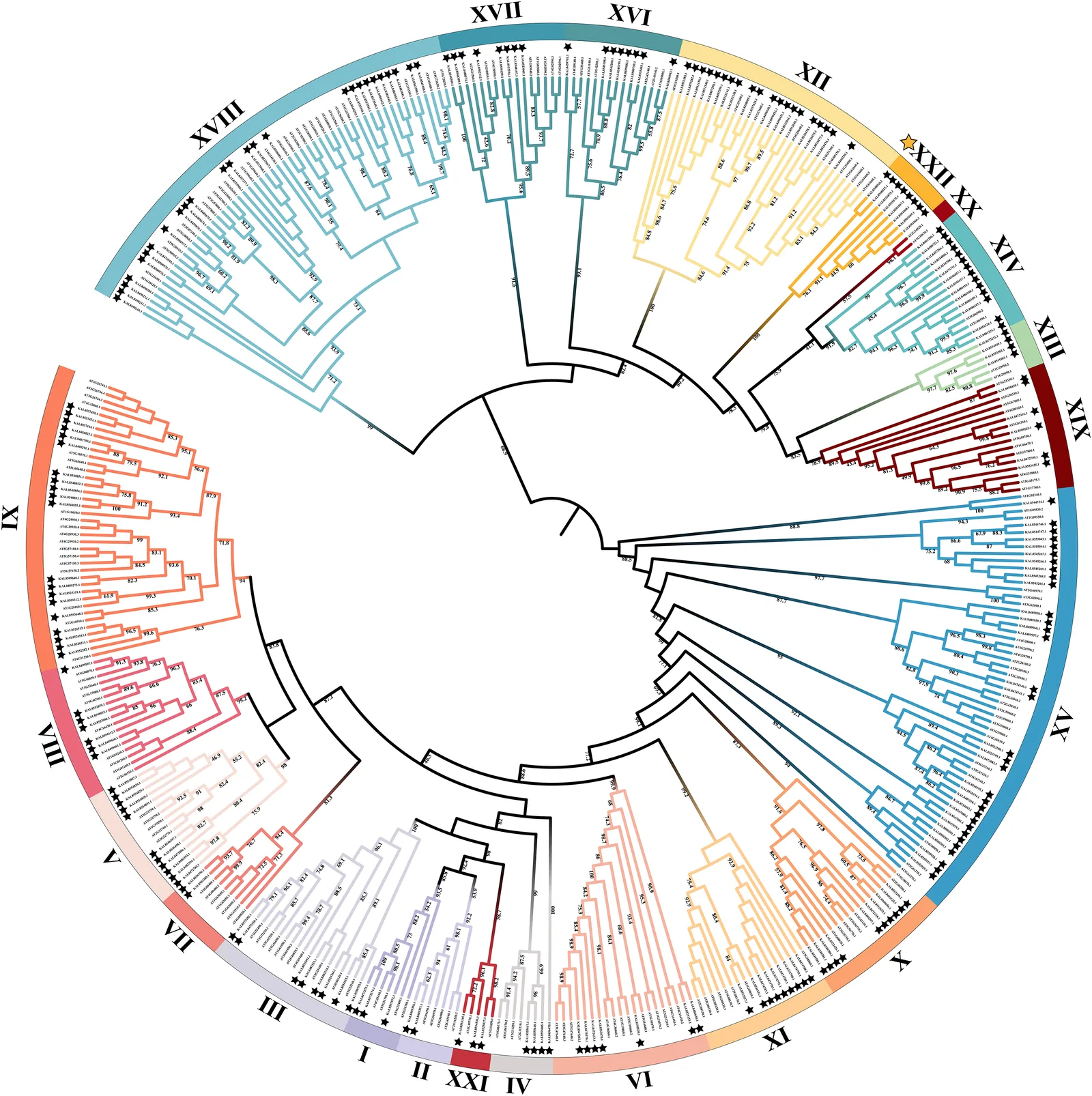
Phylogenetic reconstruction of bHLH TF genes in *P.rotata* (★) and *A. thaliana*. Bootstrap values > 40% are shown at the corresponding nodes. The resulting phylogeny resolved the bHLH TF into 21 distinct clades.

Chromosomal mapping revealed an uneven distribution of AP2/ERF and bHLH genes across the 11 chromosomes of *P. rotata* (**Supplementary Figure 2**; **Supplementary Table 10**). For the AP2/ERF family, chromosomes CM123327.1 and CM123328.1 each harbored 27 genes, while CM123329.1 and CM123332.1 each contained 20 genes. For the bHLH family, chromosomes CM123328.1, CM123327.1, and CM123333.1 contained 38, 34, and 31 genes, respectively. Notably, genes from both families frequently formed localized clusters on specific chromosomes, indicative of tandem duplication events.

Intraspecific collinearity analysis identified 31 collinear gene pairs involving 59 AP2/ERF genes and 28 collinear pairs involving 49 bHLH genes (**Supplementary Figure 3**; **Supplementary Table 11**). Analysis of duplication modes revealed that dispersed duplication was the predominant mechanism in both families, followed by whole-genome or segmental duplication (**Supplementary Table 12**). Specifically, the AP2/ERF family comprised 68 dispersed, 57 whole-genome or segmental, 22 tandem, and 8 proximal duplicates. The bHLH family consisted of 70 dispersed, 49 whole-genome or segmental, 5 tandem, 4 proximal, and 4 single-copy genes. These results indicate that dispersed and whole-genome/segmental duplications were the major drivers of AP2/ERF and bHLH family expansion in *P. rotata*.

### 3.5 Bioinformatic characterization of differentially expressed AP2/ERF and bHLH TFs

The predicted physicochemical properties of the differentially expressed AP2/ERF and bHLH TFs are summarized in **Supplementary Table 9**. Protein length varied considerably in both families, ranging from 125 to 573 aa in AP2/ERF and from 98 to 720 aa in bHLH, with molecular weight showing a corresponding broad distribution. Most proteins were acidic, accounting for 67.61% (48/71) of AP2/ERF members and 70.00% (35/50) of bHLH members. Instability index analysis classified the majority of AP2/ERF (91.5%, 65/71) and bHLH (98.0%, 49/50) proteins as unstable. Aliphatic index values suggested that all AP2/ERF proteins were relatively hydrophilic, whereas 92.00% (46/50) of bHLH proteins were hydrophilic and 8.00% (4/50) were relatively hydrophobic. Both families were predicted to localize predominantly to the nucleus.

MEME analysis identified 10 conserved motifs across the 121 proteins, with members of the same phylogenetic clade exhibiting similar motif compositions. In the AP2/ERF family, 98.60% (70/71) of proteins contained both Motif 1 and Motif 2, constituting the core conserved module, while 83.10% (59/71) also contained Motif 7 (**Supplementary Figure 4**). Specific domain architectures aligned with subfamily classifications: KAL8502506.1, KAL8549395.1, and KAL853340.1 harbored both B3 and AP2 domains (RAV subfamily); KAL8546331.1, KAL8502120.1, KAL8547033.1, KAL8490208.1, KAL8550196.1, and KAL8458681.1 each contained two AP2 domains (AP2 subfamily). Additionally, AP2 subfamily members were characterized by discontinuous CDS structures and the presence of Motif 8. Regarding the bHLH family, all proteins contained the defining bHLH-like domain. Motif 1 and Motif 2 were present in 94.00% (47/50) and 88.00% (44/50) of members, respectively, establishing these as the core conserved modules for this family (**Supplementary Figure 5**). Most bHLH genes (92.00%, 46/50) also displayed discontinuous CDS structures; notably, only KAL8480273.1 lacked identifiable UTR sequences. Phylogenetically related members consistently possessed similar conserved motif repertoires.

Analysis of the 2,000-bp upstream promoter regions identified 40 and 39 distinct cis-element types in AP2/ERF and bHLH genes, respectively, following the exclusion of core promoter elements (e.g., TATA-box). According to previous studies, these cis-acting elements were categorized into three distinct groups: abiotic and biotic stress responsiveness, phytohormone responsiveness, and plant growth and development (Mengarelli and Zanor, 2021). In AP2/ERF promoters, phytohormone-responsive elements were most prevalent (39.31%, 432/1,099), followed by stress-responsive (35.85%, 394/1,099) and growth-related elements (24.84%, 273/1,099) (**Figure 7**). Specific genes, including KAL8549064.1, KAL8458681.1, and KAL8490208.1, harbored the highest numbers of cis-elements (38, 36, and 32, respectively). Conversely, in the bHLH family, stress-responsive elements dominated (41.55%, 354/852), followed by phytohormone-responsive (33.57%, 286/852) and growth-related elements (24.88%, 212/852) (**Figure 8**). KAL8548017.1, KAL8500303.1, and KAL8501069.1 contained the highest numbers of cis-elements (45, 43, and 34, respectively). Notably, KAL8480273.1 lacked elements from all three categories and contained only Motif 2, indicating a structurally streamlined genomic organization.

**Figure 7:**
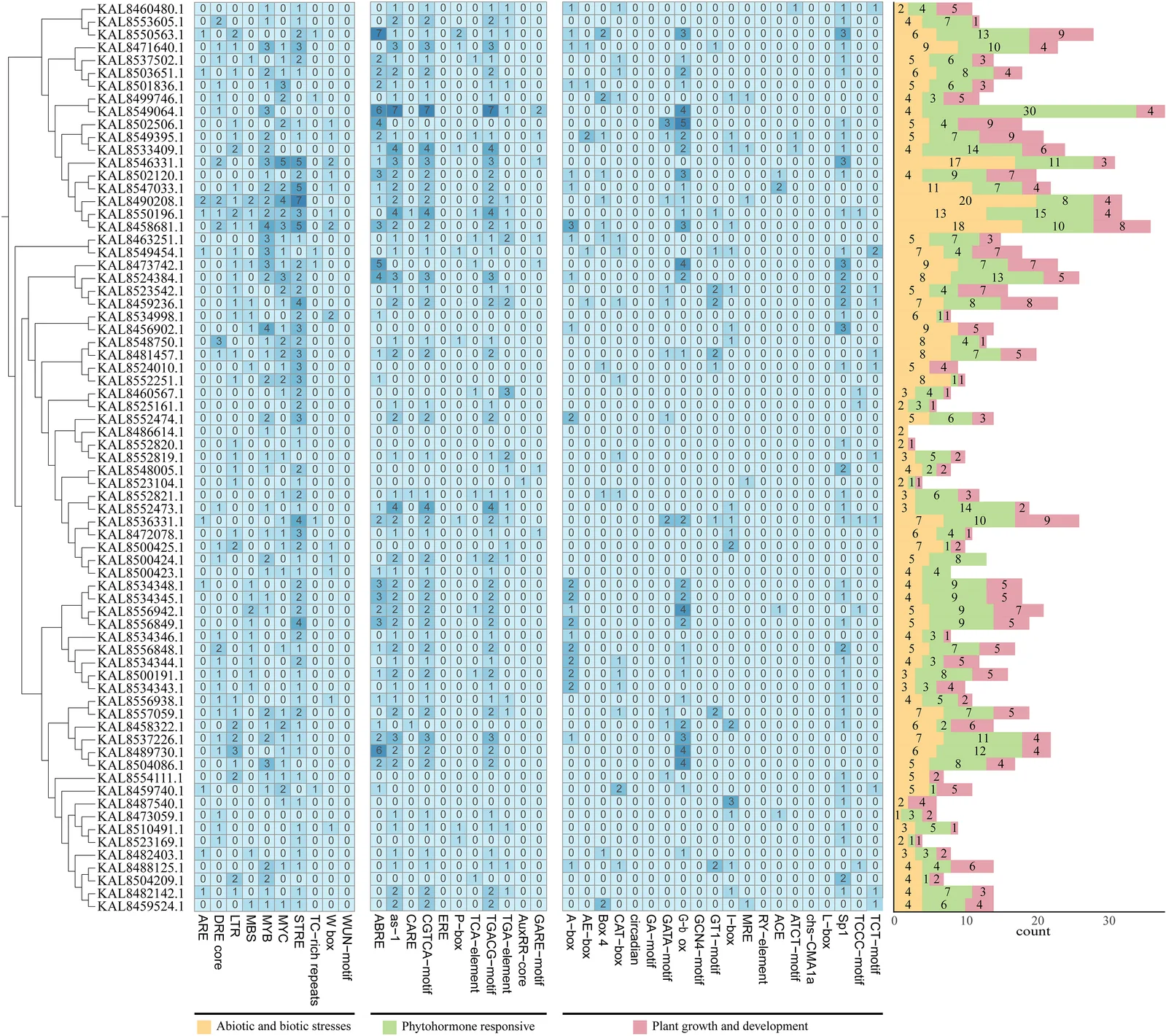
Classification of cis-regulatory elements in the promoter regions of AP2/ERF genes. Elements were categorized into three groups: abiotic and biotic stresses (yellow), phytohormone responsiveness (green), and plant growth and development (red). The detailed nomenclature of specific elements (e.g., ABRE, MYB, LTR) is provided in **Supplementary Table 13**.

**Figure 8:**
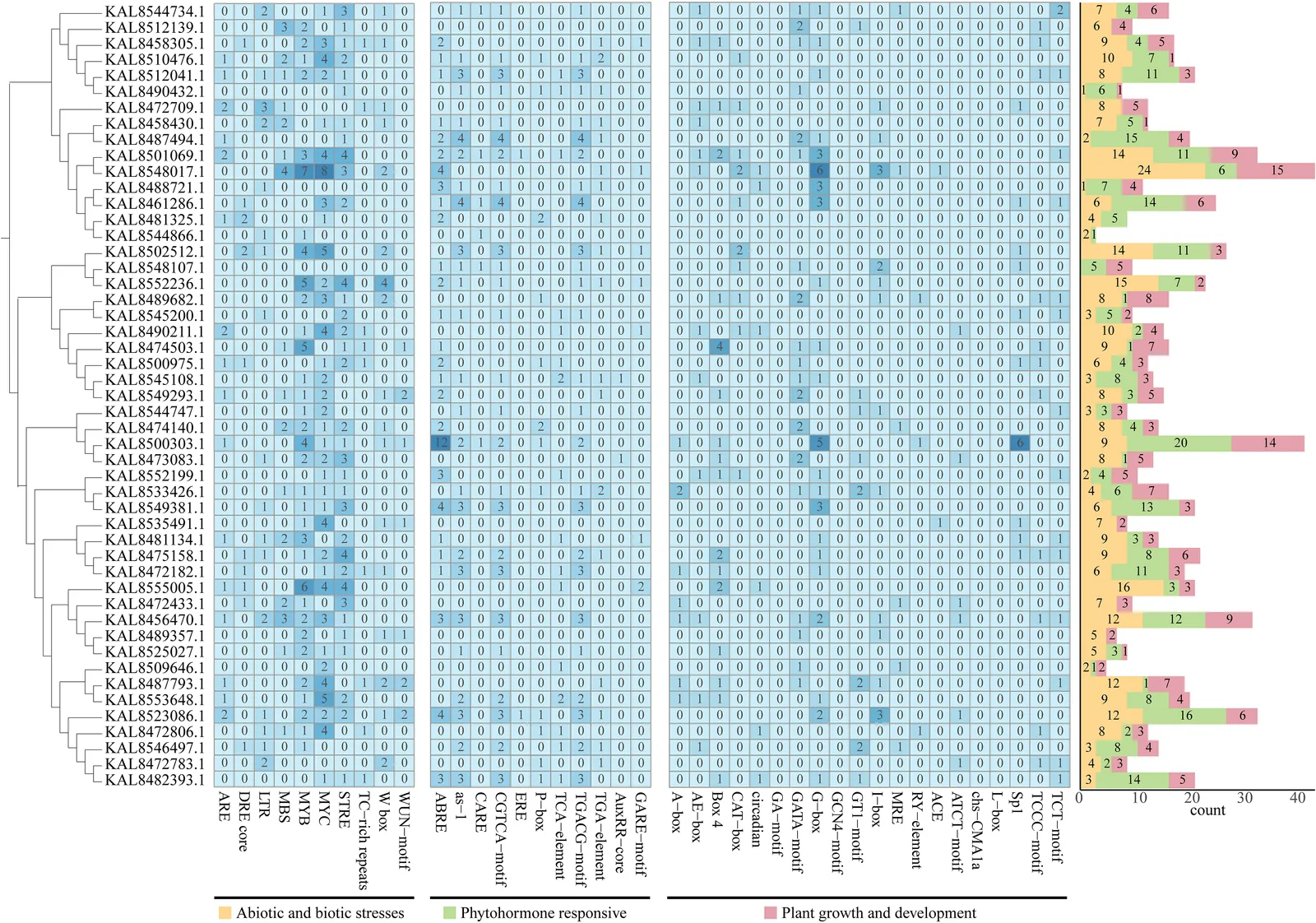
Classification of cis-regulatory elements in the promoter regions of bHLH genes. Elements were categorized into three groups: abiotic and biotic stresses (yellow), phytohormone responsiveness (green), and plant growth and development (red). The detailed nomenclature of specific elements (e.g., ABRE, MYB, LTR) is provided in **Supplementary Table 13**.

### 3.6 Co-expression network analysis of TFs, structural genes, and terpenoid metabolites

A total of 38 differentially accumulated terpenoid secondary metabolites were identified via metabolomic analysis of *P. rotata* leaves, and hierarchical clustering clearly separated samples corresponding to the four geographical populations (**Supplementary Figure 6**).

To characterize the relationships among transcription factors, structural genes, and metabolite accumulation, a weighted gene co-expression network analysis (WGCNA) was performed. The analysis integrated expression profiles of 159 target genes—comprising 71 AP2/ERF TFs, 50 bHLH TFs, and 38 structural genes—with corresponding terpenoid metabolite abundances across the 12 samples. Initial sample clustering revealed distinct groupings among biological replicates without notable outliers (**Figure 9A**), confirming high data quality. Based on the scale-free topology criterion, a soft-thresholding power of 6 was selected to construct the adjacency matrix; while the *R^2^* value approached a plateau at this threshold, it did not attain the conventional 0.85 benchmark, likely owing to the limited sample size (*n* = 12) (**Figure 9B**). Hierarchical clustering of genes was performed based on the topological overlap matrix (TOM). Dynamic tree cutting (deepSplit = 2; minimum module size = 10) followed by merging of highly similar modules (mergeCutHeight = 0.15) identified eight distinct co-expression modules, ranging in size from 11 to 30 genes (**Figure 9C**; **Supplementary Table 14**). No genes were relegated to the *gray* (unclassified) module, indicating coherent co-expression patterns within this curated gene set. To quantify the relationships between gene modules and terpenoid metabolism, Pearson correlation coefficients were calculated between each module eigengene (ME)—defined as the first principal component of gene expression within a module—and the abundance of the 38 annotated metabolites. A stringent threshold of |*r*| > 0.7 (*p* < 0.01, two-tailed t-test) was applied to identify robust module-metabolite correlations, yielding 52 significant associations across the eight modules (**Supplementary Tables 15**, **16**). Notably, the green module accounted for the largest share of these associations (12 of 52), indicating a strong correspondence between the expression profiles of its constituent genes and specific terpenoid metabolites (**Figure 10**).

**Figure 9:**
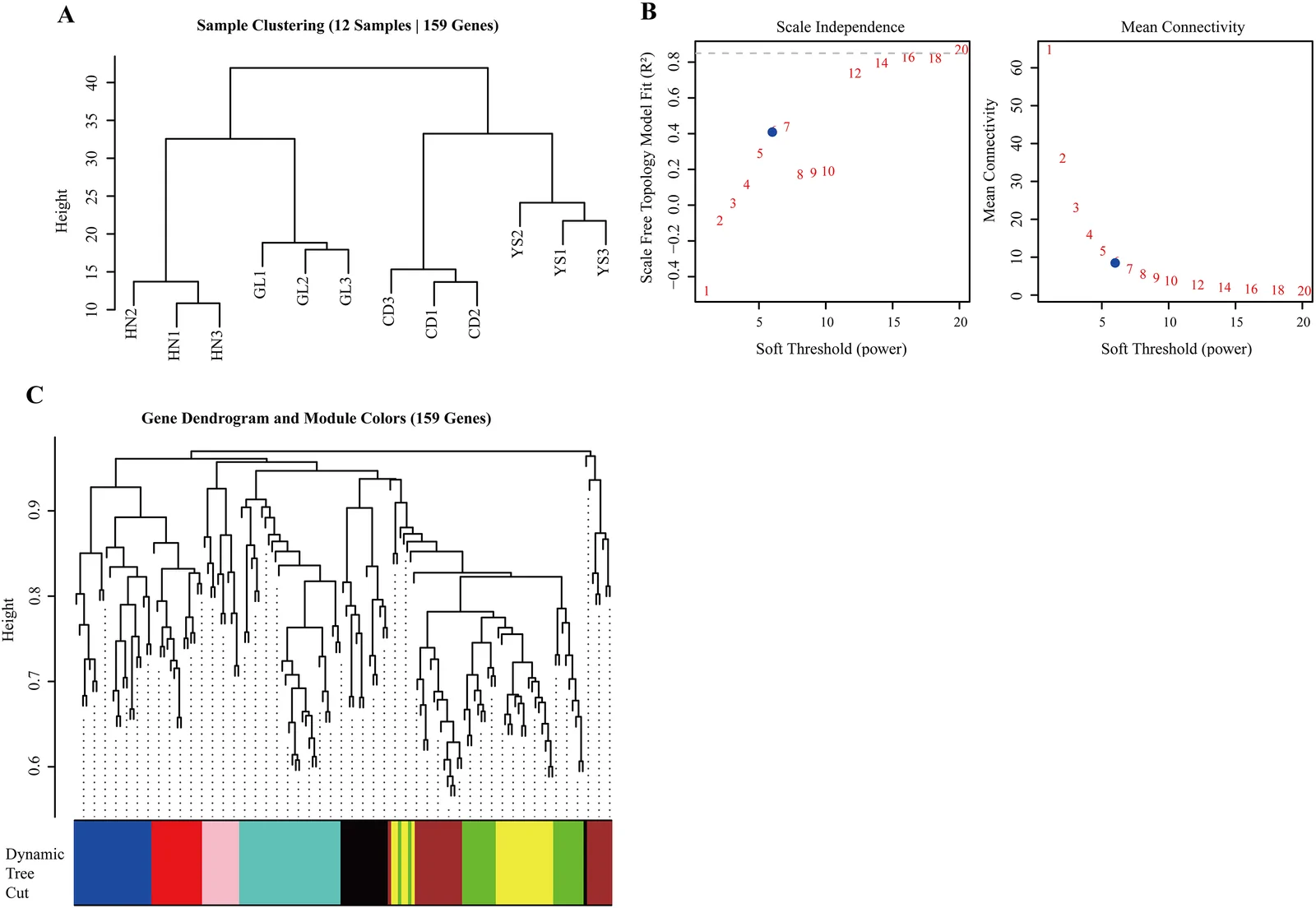
WGCNA of *P. rotata.* **(A)** Sample clustering and outlier detection. **(B)** Selection of the soft-thresholding power for gene co-expression network construction. The left panel shows changes in the scale-free topology fit index across different soft-thresholding powers. The *gray* dashed line denotes the reference value of *R^2^* = 0.85, and the blue point indicates the selected soft-thresholding power of 6. The right panel shows changes in mean connectivity across different soft-thresholding powers, with the selected value indicated by the blue point. **(C)** Module detection using a gene clustering dendrogram.

**Figure 10:**
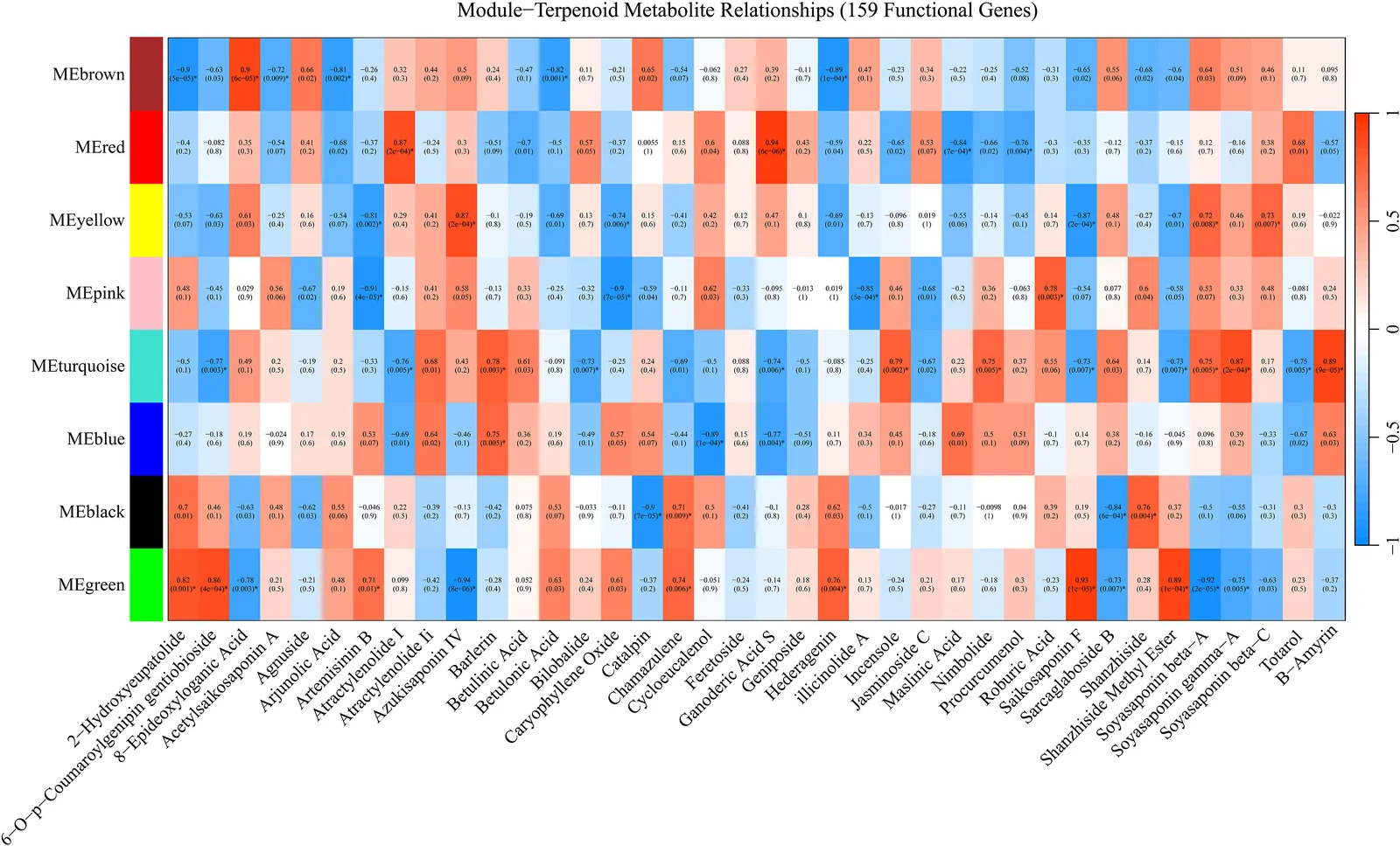
Correlation heatmap between module eigengenes and terpenoid metabolites heatmap. Rows represent module eigengenes, and columns represent terpenoid secondary metabolite traits. Each cell displays the biweight midcorrelation coefficient between the corresponding module and trait, with the associated two-sided P value shown in parentheses. The color scale ranges from blue (negative correlation) through white (no correlation) to red (positive correlation). Associations meeting the criteria for |*r*|>0.7 and *P<0.01* are marked with an asterisk (*).

To further resolve the intra-modular correlations within the green module, we performed a finer-scale analysis correlating gene expression with the levels of 12 core terpenoid metabolites (**Figure 11**). This analysis revealed distinct correlation patterns between specific TFs and metabolites. For instance, a subset of AP2/ERF and bHLH TFs (e.g., KAL8488125.1 to KAL8552474.1) exhibited strong negative correlations with Azukisaponin IV, Soyasaponin gamma-A and Soyasaponin beta-A, but strong positive correlations with 6-O-p-Coumaroylgenipin gentiobioside and Shanzhiside Methyl Ester. Conversely, other TFs (KAL8500423.1 to KAL8488125.1 and KAL8490208.1 to KAL8548005.1) showed strong positive correlations with the structural genes KAL8462524.1, KAL8555971.1, KAL8549072.1, and KAL8501460.1, whereas members of the KAL8534348.1 to KAL8456470.1 clade exhibited strong negative correlations with the same set of structural genes. Additionally, four structural genes (SGs) displayed strong positive correlations with 2-Hydroxyeupatolide, 6-O-p-Coumaroylgenipin gentiobioside, Shanzhiside Methyl Ester, Hederagenin, and Chamazulene, but strong negative correlations with Azukisaponin IV, Soyasaponin beta-A and Soyasaponin gamma-A.

**Figure 11:**
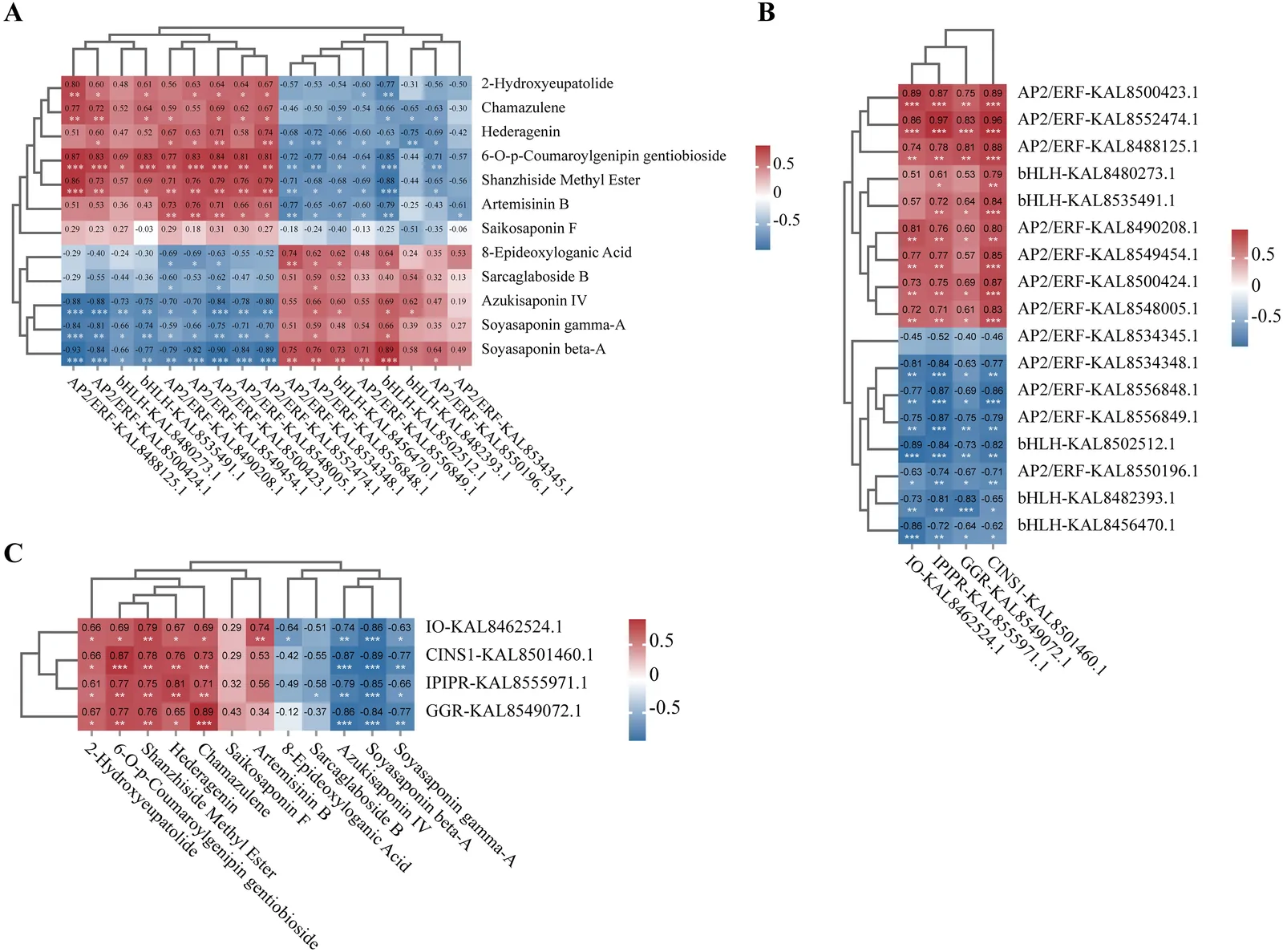
Correlation heatmap within the green module. **(A)** Correlation between TFs and metabolites. **(B)** Correlation between TFs and structural genes (SGs). **(C)** Correlation between SGs and metabolites. Red and blue indicate positive and negative correlations, respectively. Significance levels are indicated as follows: *: *P* < 0.05; **: *P* < 0.01; ***: *P* < 0.001. Labels in the heatmap incorporate a family prefix (AP2/ERF for AP2/ERF family members, bHLH for bHLH family members; applied to both transcription factors and structural genes) concatenated with the original genome annotation identifier (KALxxxxxx.1) via a hyphen, to enable straightforward visual distinction of gene classes. All gene citations in the main text exclusively use the full original annotation identifier to guarantee sequence traceability.

### 3.7 Validation of transcriptome data by qRT-PCR

Eighteen representative genes from the AP2/ERF and bHLH families were selected for validation via quantitative real-time PCR (qRT-PCR). All primer sequences are listed in **Supplementary Table S18**. The gene *TFIIS* (KAL8552379.1) was employed as an endogenous reference gene for normalizing target gene expression levels. The expression patterns of the selected 18 genes derived from qRT-PCR exhibited a high degree of congruence with the RNA-seq data, thereby confirming the reliability of the transcriptomic profiles.

Analysis of the AP2/ERF family members revealed distinct expression profiles across the four geographical populations (**Figure 12**). For instance, *ERF017*, *ERF110*, *ERF098*, and *ERF022* exhibited their highest expression levels in the HN population, while expression was comparatively lower in the GL, YS, and CD populations. Conversely, *ERF025* and *DREB2B* peaked in the GL population, whereas *RAP2–3* and *ERF114* were most highly expressed in the CD population. Similarly, within the bHLH family, members displayed heterogeneous expression patterns (**Figure 13**). *BEE3* expression was highest in the CD population, while *BHLH25* and *BHLH3*5 were most abundant in HN. *BHLH137*, *IBL1*, *LHW*, and *UNE12* showed maximal expression in GL, whereas *PIL15* peaked in YS.

**Figure 12:**
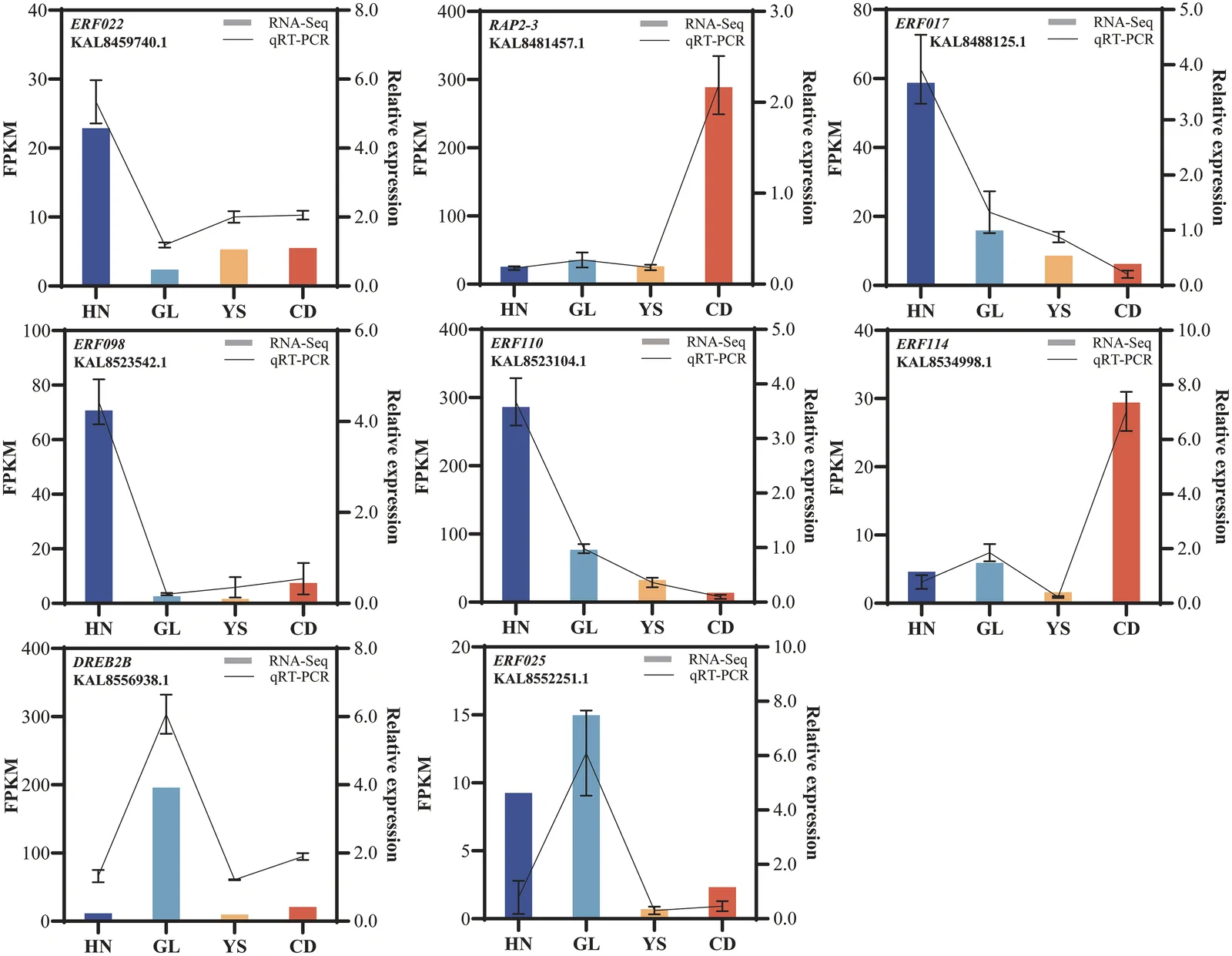
Integrated transcriptomics and qRT-PCR validation of AP2/ERF TF expression in *P. rotata* leaves from different regions. Bar charts represent the transcript abundance (FPKM) derived from RNA-seq, while line graphs illustrate the relative expression levels quantified by qRT-PCR. Data are expressed as the mean ± standard deviation (SD) derived from three independent biological replicates (n = 3).

**Figure 13:**
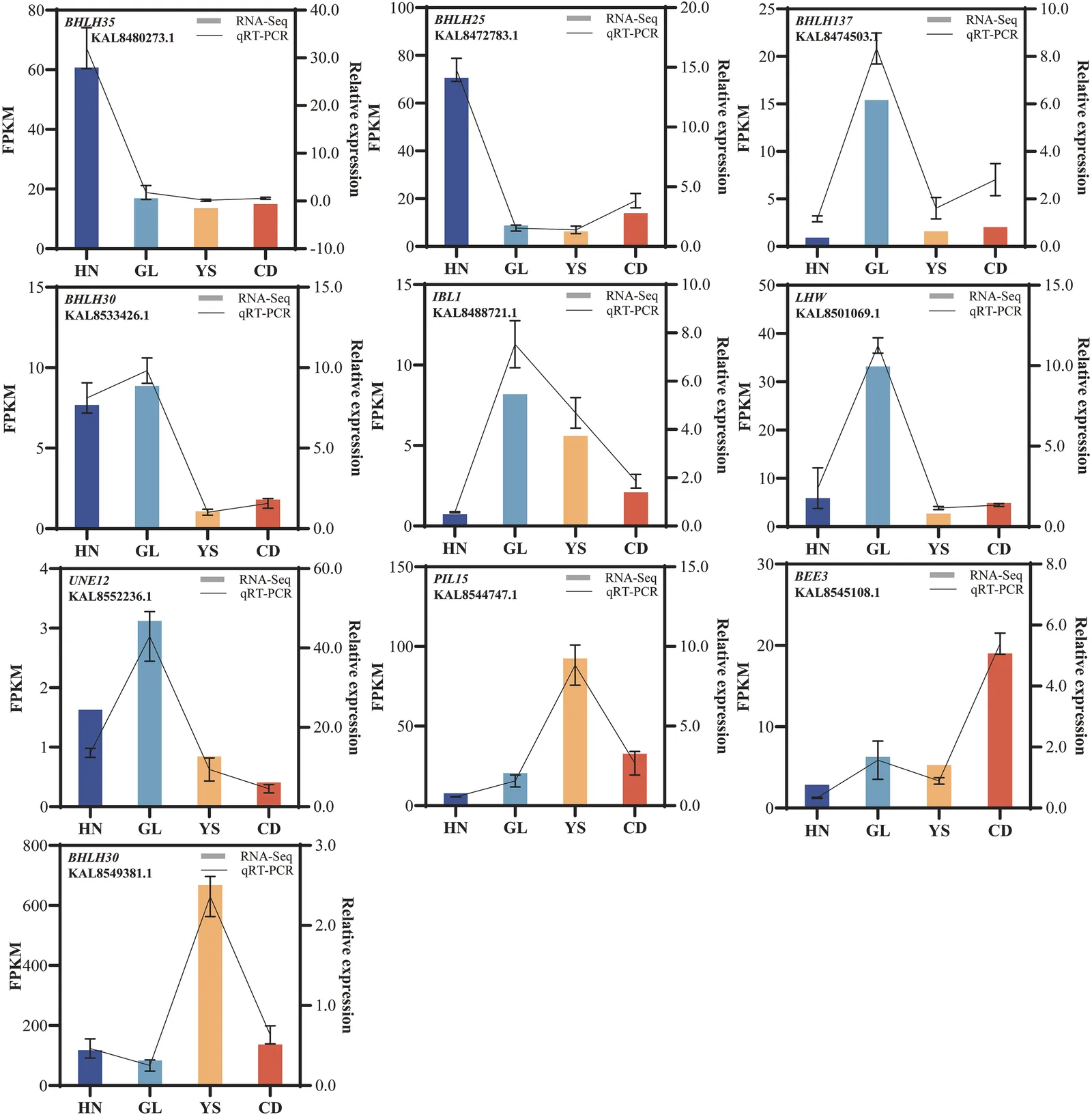
Integrated transcriptomics and qRT-PCR validation of bHLH TF expression in *P. rotata* leaves from different regions. Bar charts represent the transcript abundance (FPKM) derived from RNA-seq, while line graphs illustrate the relative expression levels quantified by qRT-PCR. Data are expressed as the mean ± standard deviation (SD) derived from three independent biological replicates (n = 3).

## 4 Discussion

In recent years, research on *P. rotata* has expanded from single-metabolite studies to a multi-omics scale. Wang et al. (Wang et al., 2024) characterized genes involved in iridoid biosynthesis in wild populations and identified key genes within the flavonoid pathway. Notably, a study by Wu et al. (Wu et al., 2025) integrated transcriptomic, metabolomic, and rhizosphere microbial data from cultivated populations spanning 1,293–3,506 m, establishing an “altitude–microbe–host–metabolism–phenotype” framework. In contrast, the present study focuses exclusively on wild populations across a higher elevational range (3,540–4,270 m), with our lowest sampling altitude exceeding the maximum altitude reported in the aforementioned cultivated populations. Sequencing data demonstrated high reliability, with uniform gene coverage (>0.9), strong intragroup correlations (up to 0.97), and clear clustering of the four geographical populations via PCA (cumulative variance 74.73%). Differential expression analysis revealed a non-linear pattern of transcriptomic divergence: the GL vs. CD comparison (520 m elevational difference) yielded 4,286 DEGs, surpassing the 3,613 DEGs detected in the HN vs. CD comparison (730 m difference). This quantitative discrepancy in DEG counts across specific population pairs highlights heterogeneous patterns of gene expression variation among wild populations. KEGG pathway analysis further revealed a distinct stratification of terpenoid biosynthetic pathways across the gradient. While monoterpenoid biosynthesis was prominently enriched in comparisons below 4,000 m, sesquiterpenoid and triterpenoid biosynthesis were exclusively enriched in lower-elevation contrasts (HN vs. YS and HN vs. GL). Crucially, in the highest-elevation contrast (YS vs. CD), no terpenoid-specific pathways remained significantly enriched, indicating a marked quantitative reduction in the transcriptional activity of specialized terpenoid pathways above ~3,900 m. This observation aligns with species-specific metabolic variation reported in other alpine plants, such as *Rhododendron sanguineum* var. haemaleum, where high-elevation populations exhibit attenuated terpenoid gene expression (Ye et al., 2023). Unlike *Incarvillea younghusbandii*, which maintains upregulated terpenoid genes up to 5,200 m (Wei et al., 2025), *P. rotata* exhibits a distinct attenuation of complex terpenoid biosynthesis at extreme altitudes, coinciding with a relative increase in pathways related to glutathione and zeatin metabolism. These altitude-dependent shifts in terpenoid transcriptional activity parallel environmental modulation of terpenoid pathways observed in other medicinal plants. For example, high-light exposure drives diterpenoid lactone biosynthesis in *Andrographis paniculata* through integrated light and salicylic acid signaling (Zhou et al., 2026), illustrating that environmental cues can reshape terpenoid metabolic networks in medicinal plants.

Although transcription factors constitute a small fraction of plant genomes, they represent key components of gene regulatory networks and have thus attracted considerable attention across diverse plant species (Tian et al., 2019; Lyu et al., 2026). Nevertheless, systematic characterization of transcription factor families in the alpine medicinal plant *P. rotata* remains limited. Variations in genome size and evolutionary history contribute to differences in the complement and copy number of TFs among species (Tian et al., 2019). For instance, the AP2/ERF family varies considerably in size, with 318 members identified in *Panax ginseng* (Wang et al., 2025b), 175 in *Codonopsis pilosula* (Zhang et al., 2026b), and 169 in *Salvia miltiorrhiza* (Chen et al., 2026b). Similarly, bHLH family membership differs, with 171 members reported in Uncaria rhynchophylla (Shao et al., 2024) and 68 in *Nothapodytes nimmoniana* (Godbole et al., 2023). In this study, genome-wide analysis of *P. rotat*a leaves identified 54 transcription factor families. Further characterization of 71 differentially expressed AP2/ERF and 50 differentially expressed bHLH members revealed variations in molecular weight and amino acid length between the two families. Physicochemical profiling indicated that the majority of these proteins are acidic, unstable, and predominantly hydrophilic—properties congruent with those reported for AP2/ERF in Nothapodytes nimmoniana (Zhang et al., 2024) and *Panax ginseng* (Wang et al., 2025b), as well as bHLH TFs in *Citrus sinensis* (Arshad et al., 2025) and *Glycyrrhiza uralensis* (Ding et al., 2025). Subcellular localization predictions placed these proteins within the nucleus, consistent with their canonical role as transcription factors. Phylogenetic analysis resolved the evolutionary architecture of these families in *P. rotata*. Based on the classification system in *Arabidopsis thaliana*, AP2/ERF proteins were delineated into five subfamilies (AP2, DREB, ERF, RAV, and Soloist), with DREB and ERF further classified into groups A1–A6 and B1–B6, respectively. For the bHLH family, genes were assigned to 20 of the 21 known subgroups (I–XXI). Notably, a distinct, well-supported clade comprising eight *P. rotata*-specific bHLH members was identified and designated as subgroup XXII, suggesting a lineage-specific expansion. Chromosomal mapping revealed uneven distributions and localized clustering, indicative of tandem duplications. Dispersed and whole-genome/segmental duplications were identified as the primary drivers of family expansion, a pattern consistent with observations in goji (*Lycium barbarum*) (Li et al., 2025b) and rubber tree (*Hevea brasiliensis*) (Fan et al., 2025). Promoter analysis revealed divergent cis-element compositions: hormone-responsive elements predominated in AP2/ERF promoters (39.31%), whereas stress-responsive elements were most abundant in bHLH promoters (41.55%). This divergence in cis-regulatory architecture mirrors patterns observed in quinoa (Bakhtari et al., 2024) and Rosaceae species (Zhuang et al., 2024), reflecting differential sensitivities to endogenous and exogenous cues among these TF families (Liu et al., 2024).

Transcription factors play key roles in the biosynthesis of plant secondary metabolites by regulating the expression of structural genes (Li et al., 2025a). In a variety of medicinal plants, members of the AP2/ERF and bHLH families have been shown to participate in the regulation of terpene metabolic pathways. For example, Chen et al. systematically reviewed the mechanisms by which AP2/ERF regulates terpenoid, phenolic, and alkaloid products such as artemisinin, tanshinones, and anthocyanins through GCC-box, CE1, and DRE cis-elements as well as interactions with MYB, bHLH, and WRKY factors (Chen et al., 2026a). Under MeJA induction, *MhbHLH87* in mint activates *MhND* and *MhG8H1* to promote monoterpene biosynthesis, while simultaneously repressing *MhPAL1* and *MhF3H* to reduce flux through the phenylpropanoid pathway (Wang et al., 2025a). In the present study, the green module identified by WGCNA exhibited the strongest correlations with terpenoid metabolites. Correlation analysis revealed that 17 TFs were strongly associated with 12 terpenoid metabolites; 70.59% (12/17) belonged to AP2/ERF and 29.41% (5/17) belonged to bHLH. This distribution aligns with previous reports identifying AP2/ERF and bHLH as prominent families in terpenoid biosynthesis (Yang et al., 2022; Chen et al., 2026a).

To further explore the correlative basis of module–metabolite associations, we analyzed correlations between genes in the green module and 12 core terpenoid metabolites. Distinct correlation patterns were observed among AP2/ERF and bHLH family members. Certain AP2/ERF transcripts (KAL8488125.1 to KAL8552474.1) negatively correlated with triterpenoid saponins (azukisaponin IV, soyasaponin γ-A, soyasaponin β-A) but positively correlated with iridoid glycosides (6-O-p-coumaroylgenipin gentiobioside, shanzhiside methyl ester). The genes *IO*, *IPIPR*, *GGR*, and *CINS1* are essential for terpenoid skeleton formation and determine the availability and directional flux of isoprenoid precursors (Vranová et al., 2013). In the green module, AP2/ERF and bHLH members displayed divergent correlation patterns with these structural genes. Such antagonistic correlation patterns within individual TF families are a conserved metabolic strategy in plants. For instance, bHLH paralogs exert opposing effects on artemisinin biosynthesis in *Artemisia annua* (Shen et al., 2022), and differential ERF subfamily members positively or negatively modulate terpene synthase expression in *Litsea cubeba* and *Dendrobium officinale* (Wang et al., 2022; Li et al., 2024a). Moreover, in our study *IO*, *IPIPR*, *GGR* and *CINS1* structural genes were positively correlated with iridoids, monoterpenoids, and hederagenin (2-hydroxyeupatolide, 6-O-p-coumaroylgenipin gentiobioside, shanzhiside methyl ester, chamazulene), but negatively correlated with triterpenoid saponins (azukisaponin IV, soyasaponin β-A, soyasaponin γ-A). It is speculated that these four structural genes may be involved in terpenoid biosynthesis.

In summary, this study provides a comprehensive genomic and transcriptomic characterization of AP2/ERF and bHLH families in wild *P. rotata* populations across a broad elevational gradient. These findings offer a valuable comparative framework for understanding the molecular diversity of this important medicinal resource and provide candidate genes for future functional validation in related Lamiaceae species.

## 5 Conclusion

In this study, we identified 54 transcription factor families in the leaves of P. rotata from four different regions at altitudes ranging from 3,540 m to 4,270 m. AP2/ERF and bHLH were the two most abundant differentially expressed transcription factor families, comprising 71 and 50 members, respectively. We analyzed the phylogenetic relationships, chromosomal distribution, and intraspecific synteny of the genome-wide identified AP2/ERF and bHLH families. Conserved motifs, domains, gene structures, physicochemical properties of proteins, subcellular localization, and cis-acting elements were further analyzed for the differentially expressed AP2/ERF and bHLH members. By integrating multi-omics data using weighted gene co-expression network analysis (WGCNA), this study systematically investigated the molecular framework through which the AP2/ERF and bHLH transcription factor families associate with terpenoid secondary metabolism in P. rotata. With a sample size of n = 12, a soft threshold of 6 was selected to balance scale-free topology and network connectivity. The samples clustered well and no outliers were detected, supporting the reliability of the network construction. The results indicated that the green module was the core functional unit associated with terpenoid metabolism, in which AP2/ERF and bHLH members acted as central nodes. These TFs showed positive correlations with key structural genes (IO, KAL8462524.1; IPIPR, KAL8555971.1; GGR, KAL8549072.1; CINS1, KAL8501460.1) and with the accumulation of bioactive iridoid and monoterpenoid compounds (e.g., shanzhiside methyl ester), while exhibiting negative correlations with triterpenoid saponin biosynthesis. These opposing correlation patterns define the correlative framework through which AP2/ERF and bHLH networks differentially associate with iridoid/monoterpenoid accumulation versus triterpenoid saponin biosynthesis.

## Data Availability

The datasets presented in this study can be found in online repositories. The names of the repository/repositories and accession number(s) can be found in the article/**Supplementary Material**.
