# Supplementary material for: Characterization of AP2/ERF and bHLH transcription factors and association with terpenoid metabolism in Phlomoides rotata from the Qinghai-Tibet Plateau
Source: Front Plant Sci. 2026 Sep 23;17:1957015. doi: 10.3389/fpls.2026.1957015 (PMC13642777; doi:10.3389/fpls.2026.1957015)
Supplement: Supplementary file 1 [file DataSheet1.pdf]

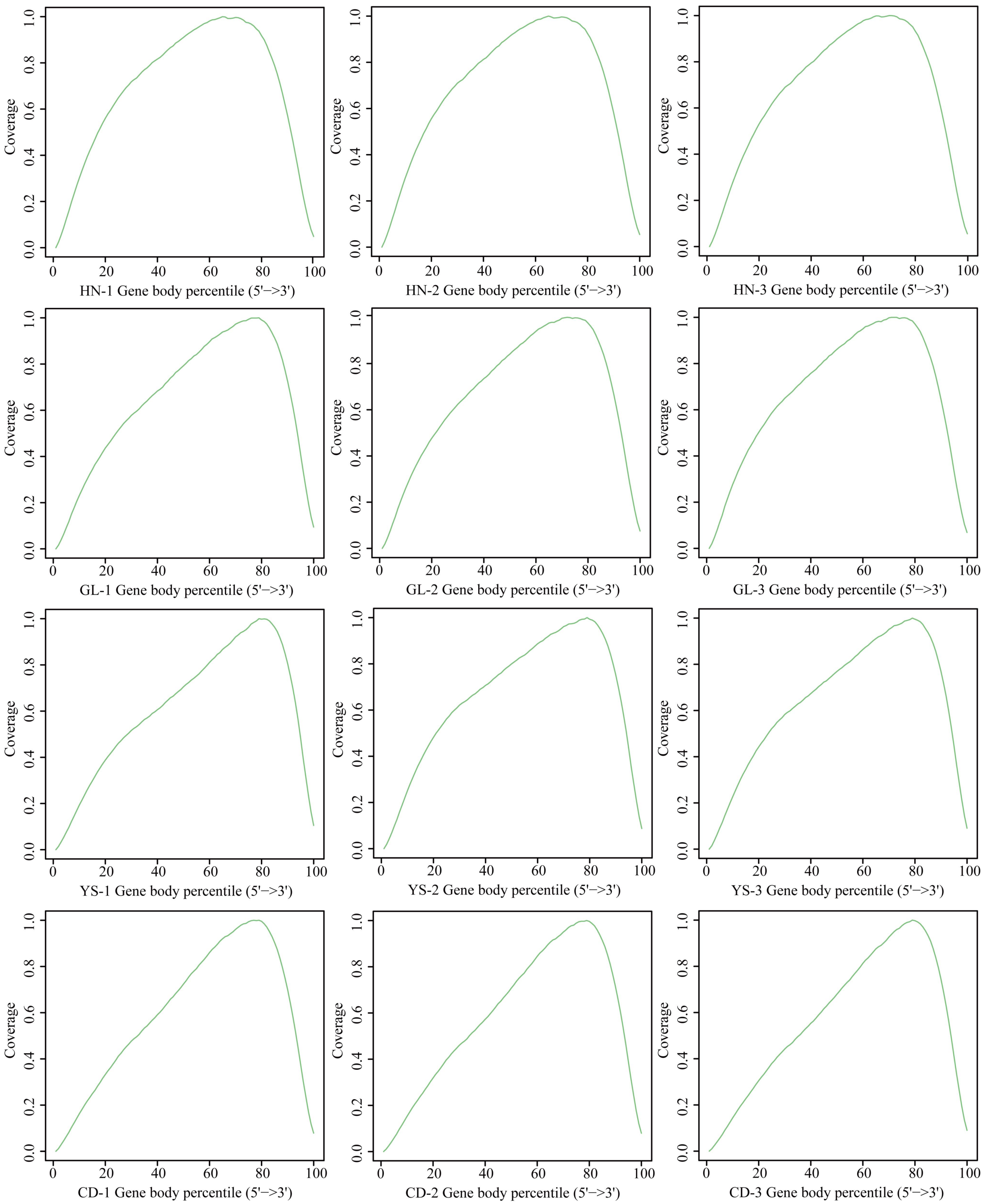

Supplementary Figure 1. Genomic region distribution of leaf transcriptome reads in *P. rotata* from

four sites at elevations ranging from 3,540 to 4,270 m. HN: 3,540 m; GL: 3,750 m; YS: 3,880 m; CD: 4,270 m.

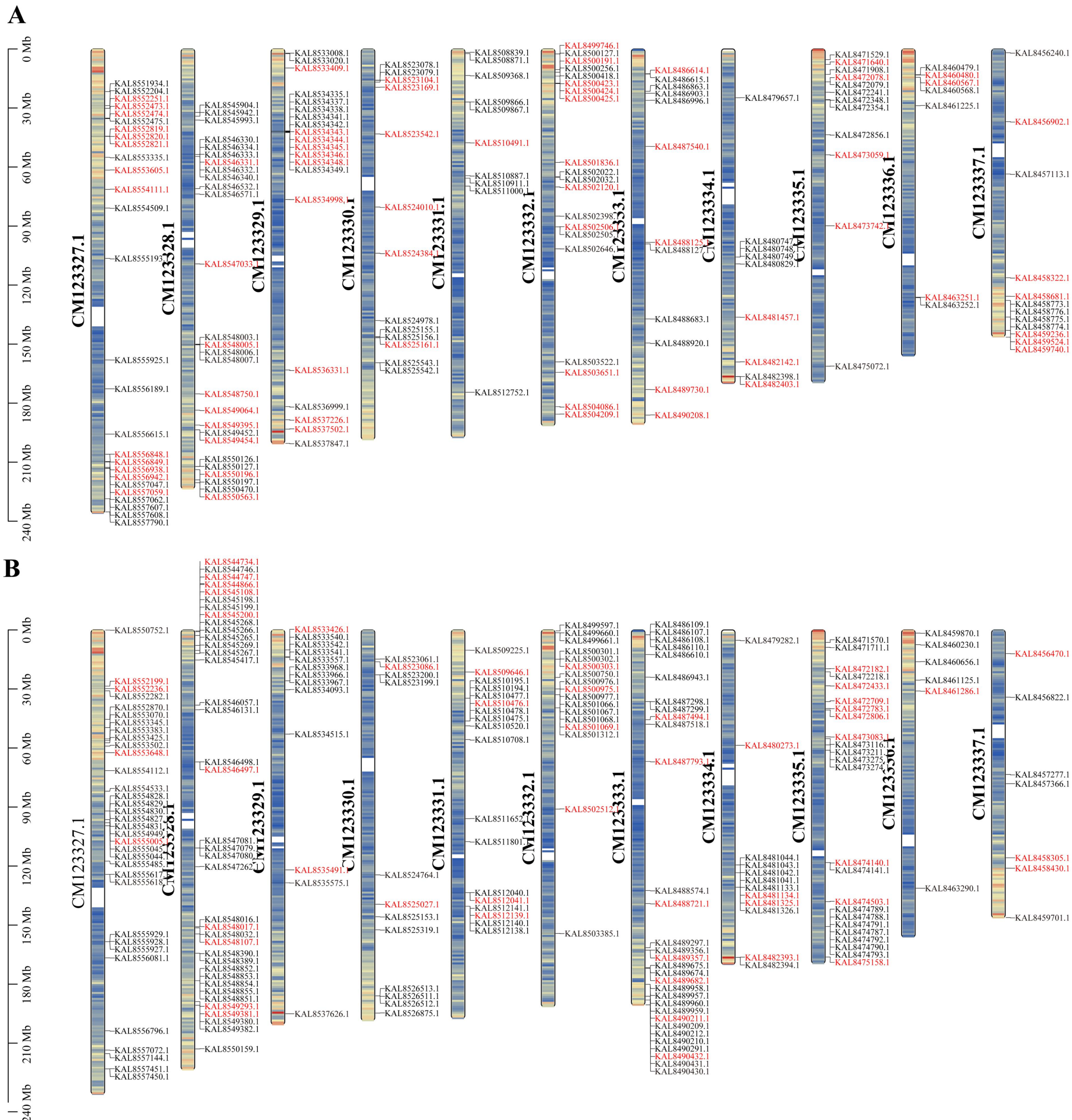

Supplementary Figure 2. Chromosomal localization analysis of AP2/ERF and bHLH transcription factors. (A) AP2/ERF. (B) bHLH. The differentially expressed genes are highlighted in red.

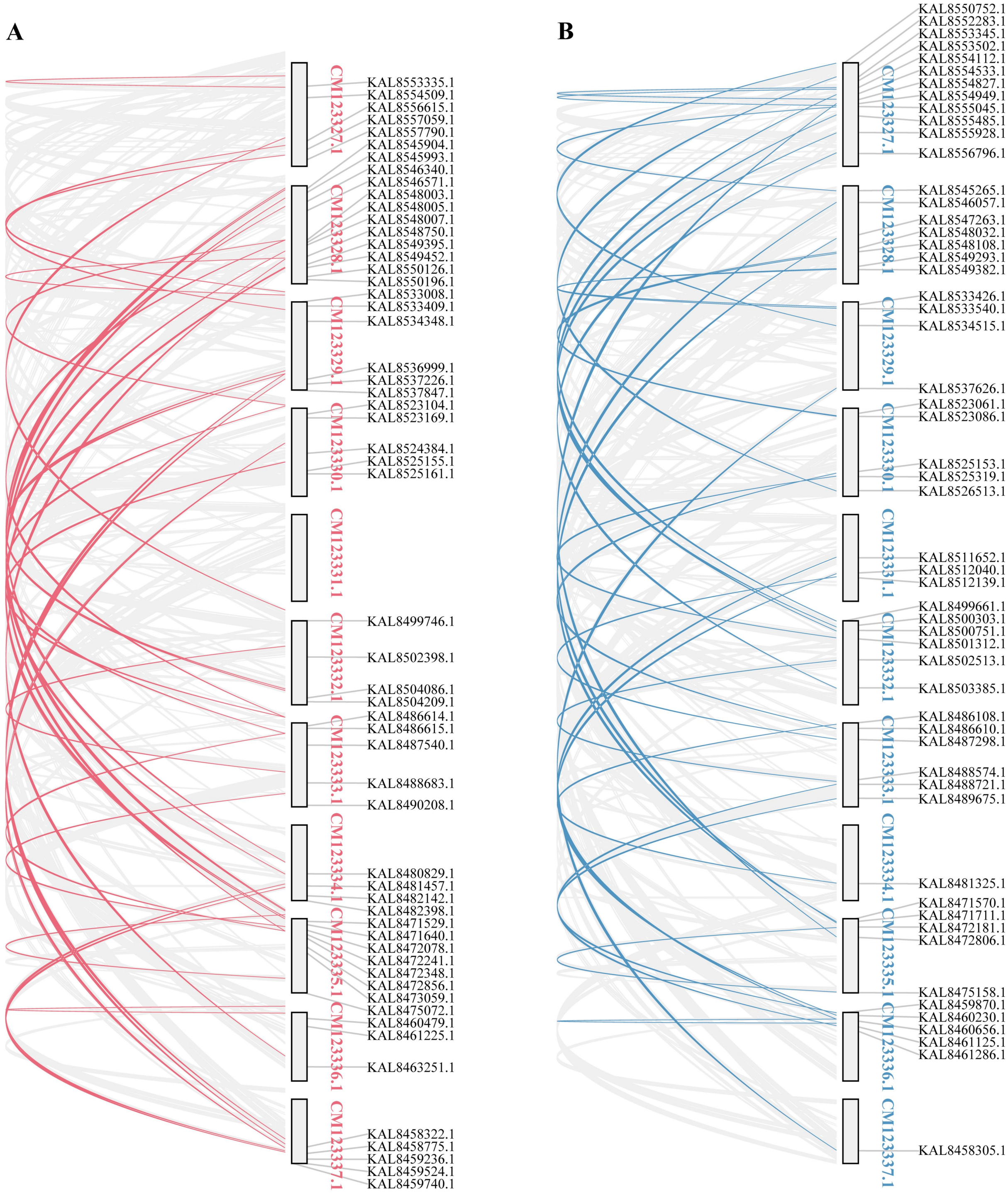

Supplementary Figure 3. Intraspecific Collinearity of AP2/ERF and bHLH Genes. (A) AP2/ERF. (B) bHLH.

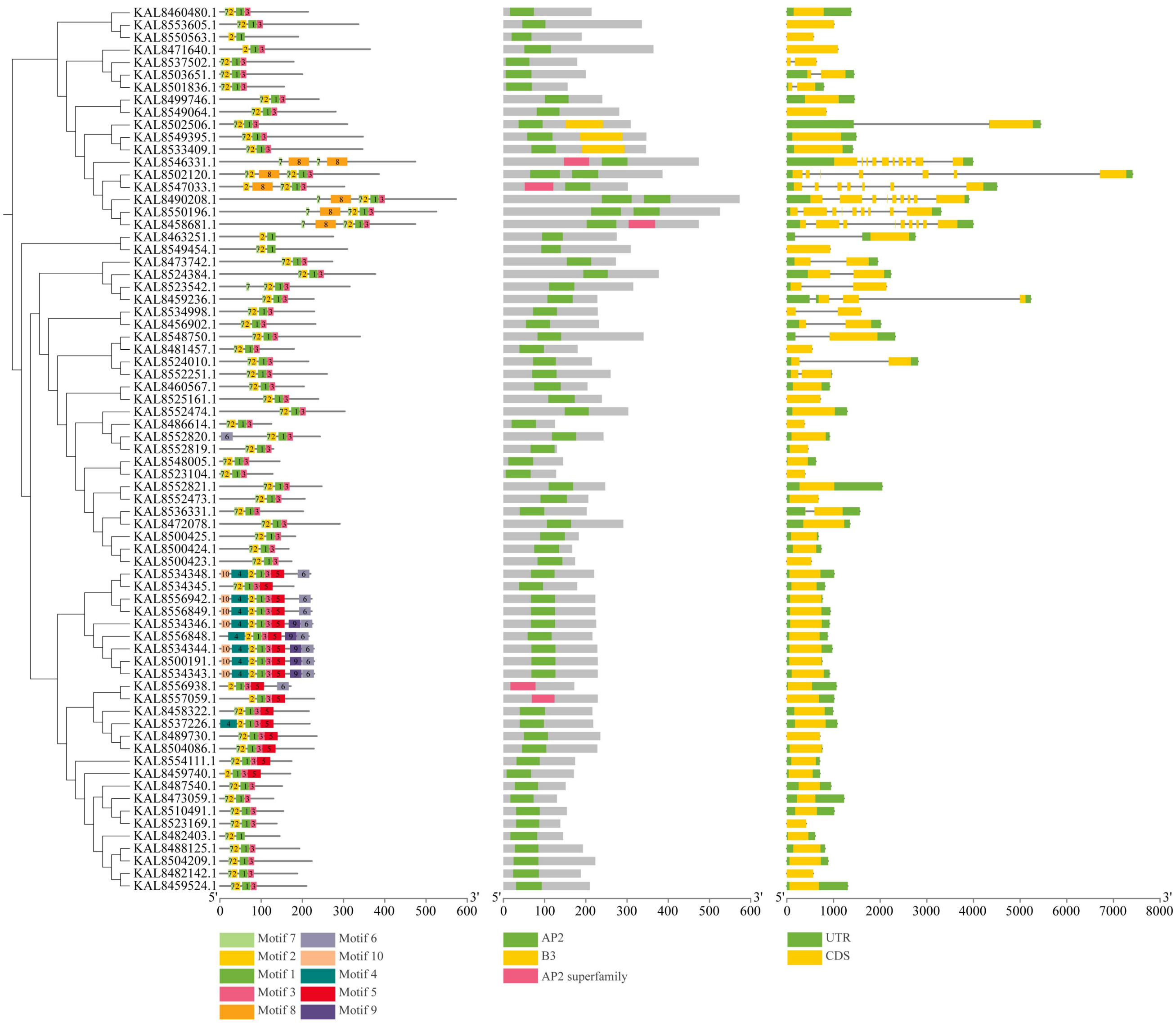

Supplementary Figure 4. Integrated Genomic Characterization of the AP2/ERF TFs in *P. rotata*: Phylogeny, Conserved Motifs, Domain Architecture, and Gene Structure.

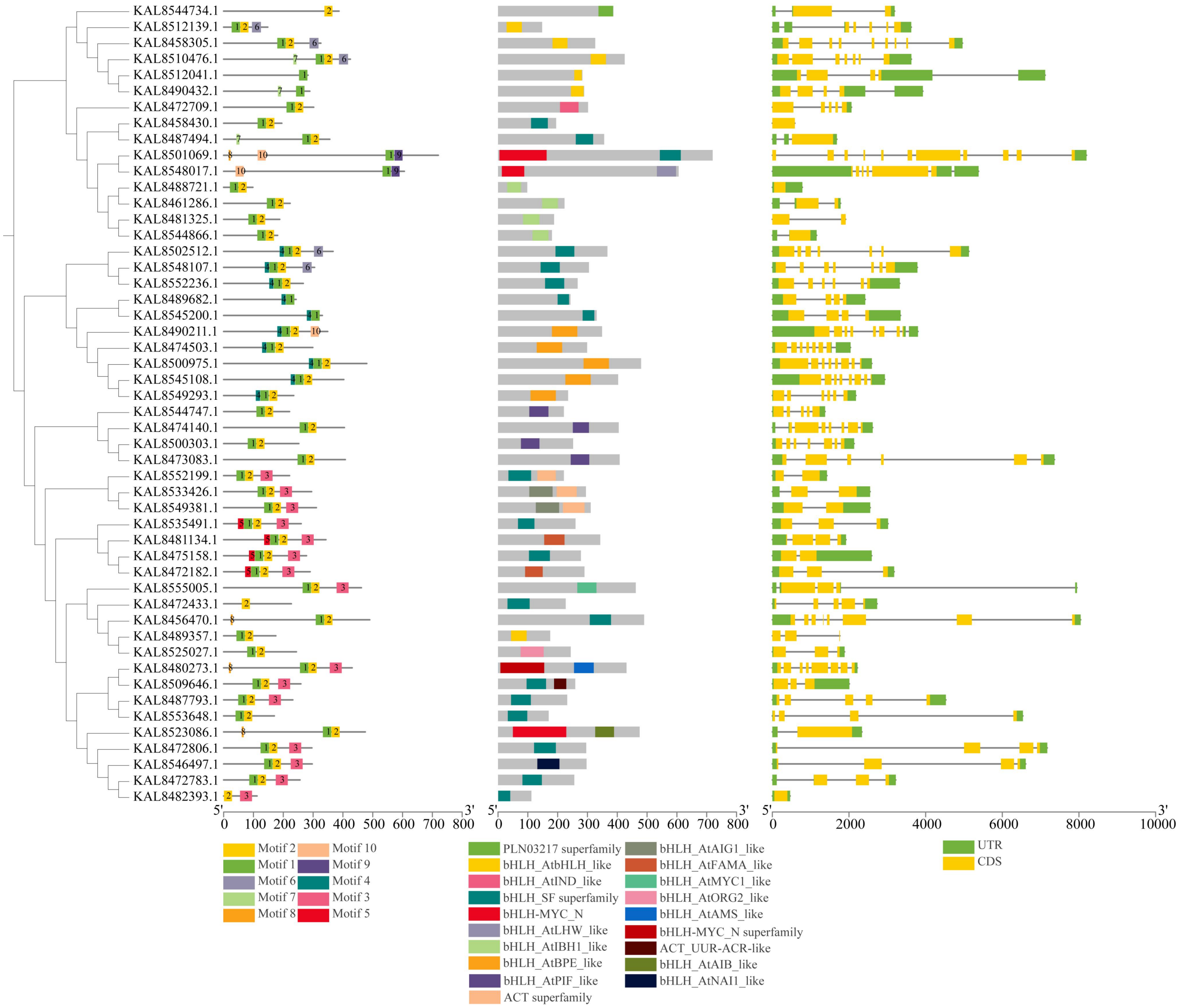

Supplementary Figure 5. Integrated Genomic Characterization of the bHLH TFs in *P. rotata:* Phylogeny, Conserved Motifs, Domain Architecture, and Gene Structure.

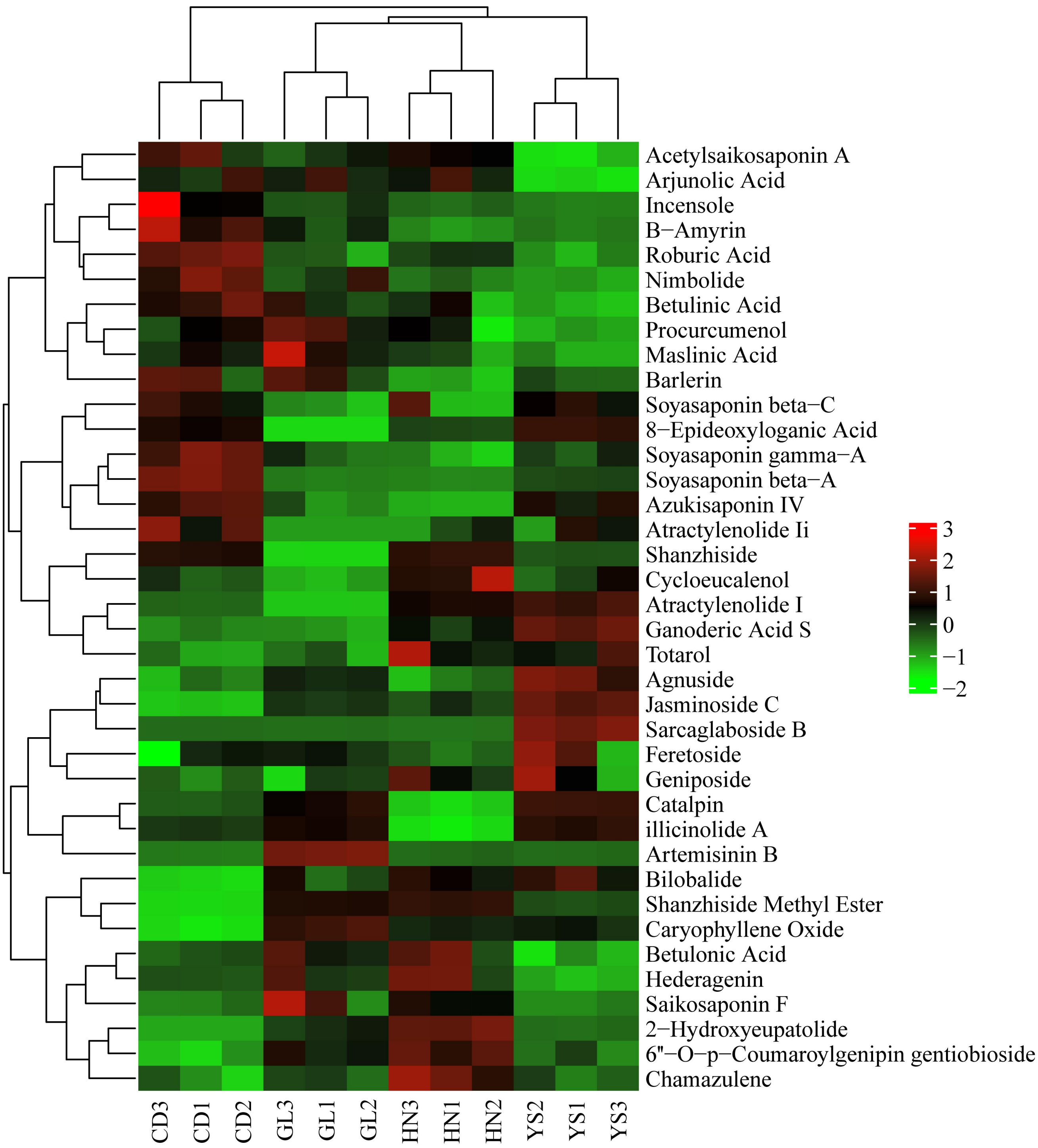

Supplementary Figure 6. Heatmap of expression levels of terpenoid secondary metabolites in *P. rotata* leaves.
